# Health Apps for Combating COVID-19: Descriptive Review and Taxonomy

**DOI:** 10.2196/24322

**Published:** 2021-03-02

**Authors:** Manal Almalki, Anna Giannicchi

**Affiliations:** 1 Department of Health Informatics Faculty of Public Health and Tropical Medicine Jazan University Jazan Saudi Arabia; 2 Berkeley College New York, NY United States

**Keywords:** app, COVID-19, corona, self-care, personal tracking, review, mHealth, track, surveillance, awareness, exposure, consumer health informatics

## Abstract

**Background:**

Mobile phone apps have been leveraged to combat the spread of COVID-19. However, little is known about these technologies’ characteristics, technical features, and various applications in health care when responding to this public health crisis. The lack of understanding has led developers and governments to make poor choices about apps’ designs, which resulted in creating less useful apps that are overall less appealing to consumers due to their technical flaws.

**Objective:**

This review aims to identify, analyze, and categorize health apps related to COVID-19 that are currently available for consumers in app stores; in particular, it focuses on exploring their key technical features and classifying the purposes that these apps were designed to serve.

**Methods:**

A review of health apps was conducted using the PRISMA-ScR (Preferred Reporting Items for Systematic Reviews and Meta-Analyses Extension for Scoping Reviews) guidelines. The Apple Store and Google Play were searched between April 20 and September 11, 2020. An app was included if it was dedicated for this disease and was listed under the health and medical categories in these app stores. The descriptions of these apps were extracted from the apps’ web pages and thematically analyzed via open coding to identify both their key technical features and overall purpose. The characteristics of the included apps were summarized and presented with descriptive statistics.

**Results:**

Of the 298 health apps that were initially retrieved, 115 met the inclusion criteria. A total of 29 technical features were found in our sample of apps, which were then categorized into five key purposes of apps related to COVID-19. A total of 77 (67%) apps were developed by governments or national authorities and for the purpose of promoting users to track their personal health (9/29, 31%). Other purposes included raising awareness on how to combat COVID-19 (8/29, 27%), managing exposure to COVID-19 (6/29, 20%), monitoring health by health care professionals (5/29, 17%), and conducting research studies (1/29, 3.5%).

**Conclusions:**

This study provides an overview and taxonomy of the health apps currently available in the market to combat COVID-19 based on their differences in basic technical features and purpose. As most of the apps were provided by governments or national authorities, it indicates the essential role these apps have as tools in public health crisis management. By involving most of the population in self-tracking their personal health and providing them with the technology to self-assess, the role of these apps is deemed to be a key driver for a participatory approach to curtail the spread of COVID-19. Further effort is required from researchers to evaluate these apps’ effectiveness and from governmental organizations to increase public awareness of these digital solutions.

## Introduction

In response to the COVID-19 pandemic, a global health movement developed with countrywide campaigns providing health education. This information was widespread to educate the public on the newly discovered SARS-CoV-2 virus and how to best protect themselves. These campaigns were filled with a series of prevention protocols and control interventions to contain COVID-19, such as social distancing, keeping infected individuals isolated, self-isolating in homes or hotels after coming into contact with someone who has tested positive, and restricting travel [1].

During the COVID-19 pandemic, health mobile phone apps have been widely used for supporting these campaigns’ missions, to assist in raising awareness on how the population may protect itself, and for encouraging adherence to those various precaution protocols [2,3]. For example, the United Kingdom National Health Service created an app with the purpose of encouraging users to keep a safe distance from others and alert them if they come close to someone who had been diagnosed with COVID-19. Within days, the app was downloaded over 10 million times, with six million downloads on the first day [4]. Globally, the World Health Organization (WHO) Alert app, a messaging service provided via social media outlets Facebook and WhatsApp that can be accessed in 15 different languages to answer questions about COVID-19, has the potential to reach two billion people [5].

In addition, health apps are not only limited to simply providing information about COVID-19 but also used to facilitate data-driven disease surveillance, screening, triage, diagnosis, and monitoring by governments or health officials, health care professionals, and health organizations [3,6]. These interventions enable timely preventative methods and treatment procedures at the population level [7]. For example, an app named COOPERA is used to monitor trends in COVID-19 in Japan, evaluate the current Japanese epidemiological situation, and provide useful insights to assist political decisions to tackle the epidemic. COOPERA collects personal information about the users and symptoms related to COVID-19. The reported number of confirmed infected cases are then calculated to detect and manage infectious cases.

At the individual level, as these health apps’ popularity rises, the opportunity has increased for consumers and patients to self-manage both their risk of exposure and symptom progression [8,9]. In combatting COVID-19, health self-management includes keeping safe distance from others to decrease the communal spread of the disease, completing self-assessments that monitor symptom development or augmentation, routinely taking prescribed medication, and maintaining a healthy diet and physical activity [1,10]. In instances such as these, mobile apps can promote health self-care practice and activate a person’s responsibility and accountability for preventing disease and maintaining health [9,11,12]. They can log and view the history of their health status, set useful reminders for treatment adherence, and provide vital information about their health status to the community to prevent future exposure.

Furthermore, the importance of health apps related to COVID-19 arises from their capabilities to allow consumers to feel safe and informed in making decisions regarding their health. For instance, an app developed by the University of California, San Francisco [13] for health care workers at the university’s hospital assesses their potential COVID-19–related symptoms. This app helps the employees avoid long queues at screening points before each clinical shift, allows for physical distancing at hospital entrances, and prevents employees with suspected cases from coming to work. Moreover, it allowed the users to answer questions about symptoms they were experiencing, such as fever, cough, and difficulty breathing. The user can then self-assess their severity and make decisions about the potential need to seek further medical treatment [14].

To ensure that apps such as these can meet consumers’ needs and preferences, it is imperative that app developers understand the various usages of these technologies and their technical features [8,15]. However, few studies have been conducted to describe COVID-19–related apps and analyze their technical features [15,16]. The lack of this understanding led developers and governments to make poor choices about health apps’ designs, which led to creating less useful apps that are overall less appealing to consumers due to their technical flaws [17]. Therefore, this review aims to identify, analyze, and categorize health apps related to COVID-19 that are currently available for consumers in app stores; particularly, it focuses on exploring their key technical features and classifying the purposes that these apps were designed to serve.

## Methods

### Identification of Relevant Apps

From April 20, 2020, to September 11, 2020, the authors explored the Apple Store and Google Play. The following search strings were used to find apps dedicated for COVID-19 that were listed under the health and medical categories: COVID19, COVID, COVID-19, corona, coronavirus, corona triage, corona symptoms, SARS-CoV-2, and respiratory diseases. Additionally, the authors searched current news articles and Google search engine results to find apps related to COVID-19 that may not have otherwise been available in the authors’ regional app store.

The app review was conducted with the PRISMA-ScR (Preferred Reporting Items for Systematic Reviews and Meta-Analyses Extension for Scoping Reviews) guidelines [18]. Although PRISMA-ScR is the standard guideline to review scientific literature, some researchers have suggested it is not completely applicable to app reviews [19,20]. Nevertheless, it has been used by several studies to review apps, as it is a common tool for performing systematic searches [19-21].

### Screening and Eligibility Assessment

The inclusion and exclusion criteria are illustrated in Table 1 and described in light of a framework developed by Ramakrishnan et al [22]. The framework encompasses a series of key questions around COVID-19–related apps that are ordered by five levels of considerations, starting from wider and going to narrower considerations as the level’s number gets higher. Although this framework was adapted from the M-Health Index and Navigation Database that has been previously used to evaluate mental health apps [22], in this study, it was used to help report our inclusion and exclusion criteria.

During the eligibility assessment round, the titles, descriptions, and keywords of identified apps were screened. Health apps that were available to the public at app stores were included. In this round, an app was excluded if it was removed from an app store by its developer during our specified search period, even if it was originally available at the beginning of this period. Their removal indicated that these apps were no longer available to consumers and were, hence, excluded from the sample.

An app was also excluded if it was only dedicated to respiratory or infectious diseases other than COVID-19, such as severe acute respiratory syndrome (SARS) or asthma without any reference to COVID-19. Examples of these apps were the Clean Your Lungs app [23], Breathcount [24], and Box Breathing [25]. Additionally, duplicated apps were identified and removed. In the event of a duplication, we included the Apple Store app, as it had all the required information about the app including its date of release.

Furthermore, no restrictions were made regarding the app’s language, pricing, store location or country of origin, developer type, or accessibility measures for gaining access to its content, such as requirement of national identification codes, local country phone numbers, or research study codes. Additionally, no restrictions were imposed in terms of the type of app users. Examples of these health apps included Spectrum [26], which is intended for clinicians to help them in making clinical decisions based on COVID-19 guidelines and infection prevention protocols; Tabaud [27], which is intended for individuals who want to know if they have been in close proximity to an infected person; Self-quarantine [28], which is intended for patients or infected people who are in self-quarantine; Covid Radar [29], which is for researchers who want to predict health care needs in specific regions; and PreWorkScreen [30], which is for employers who want to manage their employee’s COVID-19 self-screening.

**Table 1:** The inclusion and exclusion criteria of apps are presented based on a framework developed by Ramakrishnan et al [22].

Levels of consideration	Framework questions	Inclusion criteria	Exclusion criteria
App origin	Where does the app come from? Who is the developer, and what is the country of origin?	Health apps that were available at app stores during our search period	No restrictions were made on the country of origin, app’s language, or developer type.
App accessibility	On what platforms is the app available? How much does it cost, and what accessibility measures are in place for a user?	Health apps that were available in the Apple Store and Google Play	No restrictions were made on the app’s pricing or other accessibility measures for gaining access to the app’s content such as requirement of national identification codes, local country phone numbers, or research study codes.
App features	What features does the app offer, and what kind of information is it providing around COVID-19?	Health apps that were related to combating COVID-19	Health apps were excluded if they were only dedicated to respiratory or infectious diseases other than COVID-19, such as severe acute respiratory syndrome or asthma without any reference to COVID-19.
Privacy and security	Are data use and security measures specified? What kind of data are collected or shared?	Health apps that were able to collect or share data	No restrictions were made on the privacy and security measures (eg, consent forms or privacy compliance standards) or kind of data collected or shared (eg, personal information or users’ locations).
Clinical integration	For whom is it intended: patients, self-help, or essential workers?	Health apps that were available to the public	No restrictions were made on the app’s intended users.

### Selected Apps and Data Analysis

Each app’s web page in both the Apple Store and Google Play was visited. Data on each app was extracted and collected as follows: the app’s name, release date or version date when features related to COVID-19 were added, the country of origin, author or developer name, technical features, and source (link to the app’s web page). This information is summarized and presented in Multimedia Appendix 1.

The apps’ technical features were identified by performing open coding. A qualitative data analysis application (ie, Dedoose Version 8.3.35; SocioCultural Research Consultants, LLC) was used at this stage. The apps’ descriptions were first stored in a Word (Microsoft Corporation) document, which was then imported into Dedoose. Excerpts about technical features were highlighted and given a title. The code title was drawn from the excerpts’ content. For example, the excerpt “users can also engage in real-time chat with the chat feature” was placed under the title Chatbot Feature. Multimedia Appendix 2 presents the occurrence of all extracted excerpts in each app included in this review, whereas the excerpts’ content can be found in Multimedia Appendix 1 within the column technical features. After completion, the same process was repeated to identify the types of authors or developers creating these apps. Information about the authors or developers was summarized with some additional visits to their original websites to obtain more information.

**Figure 1:**
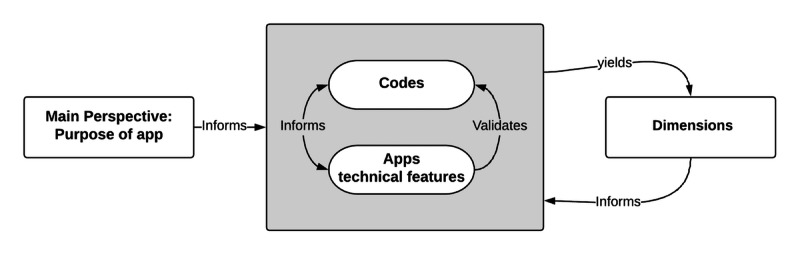
Our approach to defining purposes of apps related to COVID-19.

After this, the generated codes about the apps’ technical features were grouped into overarching categories or dimensions. Each dimension represented a different purpose that an app could serve. To identify and validate the purposes of these apps, codes and dimensions were compared iteratively to analyze the similarities in their descriptions within a category [31]. Author MA also compared the differences in codes in every other category as shown in Figure 1 (adapted from Judson et al [13]). If a code did not fit with the previously created dimensions, a new one was added. These generated dimensions were then summarized, which led to the development of our taxonomy of the apps’ purposes (as shown in the Results section).

## Results

### App Selection

**Figure 2:**
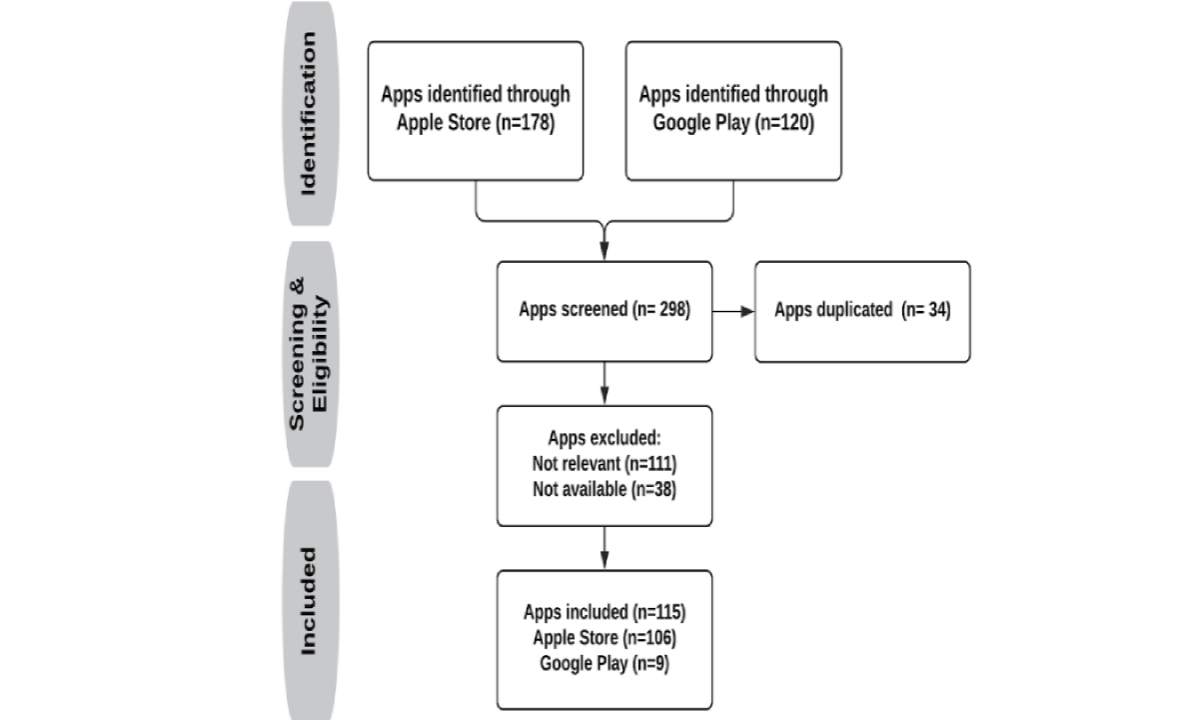
Flowchart of our search strategy and app selection process.

A total of 298 apps were identified through systematic searches in the Apple Store (n=178) and Google Play (n=120). Screening the apps’ titles and descriptions resulted in removing the following apps: 34 apps that were duplicated, 111 apps that were related to other respiratory diseases (n=56 related to SARS and n=55 related to asthma), and 38 apps that were no longer available in the app stores. After removing duplicate and irrelevant apps, 106 apps from the Apple Store and 9 from Google Play were included and further analyzed. Figure 2 illustrates the flowchart of the search strategy and app selection process.

### Characteristics of the Included Apps

The characteristics of included apps along with examples of each are presented in Table 2. Of the 115 health apps, 114 (99%) were free. Most of the apps (n=77, 67%) were developed by governments or national authorities such as the Ministry of Health in Saudi Arabia and the Government of Malaysia. Nearly one-fourth (n=25, 22%) of the apps were developed by companies such as BioneXt Lab, Paxera Health, and Webdoctor Limited. There were 5 apps from nonprofit organizations or agencies such as the WHO and Lay First Responders International. There were 4 apps built by universities or research centers such as Columbia University in the United States and the National Institute of Infectious Diseases in Romania. There were 4 apps developed by hospitals such as Merciful Brothers Hospital in Czechia.

**Table 2:** Summary of apps’ characteristics.

Characteristics		Apps (N=115), n (%)	Selected app example
**Operation system**			
	iOS	106 (92.17)	TraceCovid
	Android	9 (7.82)	Tawakkalna KSA^a^
**Pricing**			
	Free	114 (99.13)	Stop Covid19
	Not free	1 (0.87)	PreMedicus
**Developer/author**			
	Government or national authority	77 (66.95)	Covid-19 Armenia
	Company	25 (21.73)	Covive
	Nonprofit organization or agency	5 (4.35)	WHO^b^ Info
	Hospital	4 (3.48)	Trecovid19
	University or research institute/center	4 (3.48)	Covid Watcher
**The country of origin**			
	US	28 (24.35)	CovidWise
	India	5 (4.35)	AarogyaSetu
	Italy	4 (3.48)	Trecovid19
	KSA	4 (3.48)	Tabaud
	Mexico	4 (3.48)	Plan Jalisco Covid-19
	Spain	4 (3.48)	GVA CoronVirus
	Vietnam	4 (3.48)	Covid-19 Vietnam
	Global	4 (3.48)	Covive
	Canada	3 (2.61)	Canada Covid-19
	Australia	3 (2.61)	MyAus Covid-19
	France	3 (2.61)	Covidom Patient
	Netherlands	3 (2.61)	COVID-19 Medisch Dossier
	UAE	3 (2.61)	TraceCovid
	Malaysia	2 (1.74)	MyTrace
	Ireland	2 (1.74)	PatientMpower for COVID-19
	South Korea	2 (1.74)	Self-Quarantine app
	UK	2 (1.74)	NHS24 Covid-19
	Other	35 (30.43)	N/A^c^

^a^KSA: Kingdom of Saudi Arabia.

^b^WHO: World Health Organization.

^c^N/A: not applicable.

A total of 51 countries were included in this review. Most of the health apps (n=28, 24%) were from the United States, and 5 apps came from India. The following countries each created 4 apps: Italy, the Kingdom of Saudi Arabia, Mexico, Spain, and Vietnam. The following countries each made 3 apps: Australia, Canada, France, the Netherlands, and the United Arab Emirates. The following countries each developed 2 apps: Malaysia, Ireland, South Korea, Switzerland, and the United Kingdom. There were 4 apps designed for global users, and the rest came from other countries (n=35; ie, Armenia, Austria, Bahrain, Bolivia, Brazil, Columbia, Czechia, Denmark, Egypt, Georgia, Hungary, Indonesia, Iceland, Jamaica, Jordan, Kuwait, Lativa, Mali, Morocco, New Zealand, North Macedonia, Pakistan, Poland, Portugal, Qatar, Republic of Cyprus, Republic of Fiji, Romania, Singapore, Sri Lanka, Switzerland, Thailand, Tunisia, Turkey, and Uruguay).

### Technical Features of Apps Related to COVID-19

After conducting the open coding of 115 apps’ descriptions, 258 extracted excerpts were grouped into 29 technical features. Table 3 presents the key technical features with examples of apps that supported these technical features, and Multimedia Appendix 2 illustrates the occurrence of the different technical features in each app. Each technical feature is described in detail in the following sections.

The most common technical feature was *basic health information and advice* or *frequently asked questions* (*FAQs*). Over one-third (n=42, 37%) of the apps were developed to provide basic health information about COVID-19, best health practices, medical advice, and FAQs regarding COVID-19. This health information was given in the form of guidance documents, videos, and animation clips that were curated by the respective country’s government, the WHO, Centers for Disease Control and Prevention (CDC), the National Health Council, Johns Hopkins University, and other medical institutions.

The second most common technical feature in our review was *contact tracing*. Over one-fourth (n=32, 28%) of the apps supported contact tracing by documenting and retaining encounters with others such as friends, family, or coworkers. These apps allowed users to detect other devices with the same installed app and exchange an encrypted Secure Tracing Identifier (STI). The STI is stored locally on the user’s device and consists of anonymized data, a time stamp, and (in some apps) the GPS location of the phone. When any one of the users becomes infected with COVID-19, authorities with authorized access to the data can request the infected users to upload the list of STIs to their national data centers for further analysis to enable officials to respond quickly and reach out to individuals who were in close contact and who may be requested to quarantine, thus potentially minimizing the spread of the virus.

The third most common technical feature was *alert contacts*. Over one-fourth (n=30, 26%) of the apps provided the opportunity to alert contacts. These apps enabled users diagnosed with COVID-19 to voluntarily share their tests results with people they had come into contact with over the previous 14 days. These alerts were received through notifications, text messages, and automatic calls. In contrast, some apps were configured to allow health officials to automatically and anonymously inform the user’s contacts of any encounters; the users’ consents were usually obtained during the initial download of the app.

The fourth most common technical feature was *gadget of self-assessment*. There were 20 (17%) apps that provided self-assessment tools to examine whether the user may have COVID-19. These tools included questionnaires that were designed according to CDC guidelines, WHO recommendations, and the country’s health officials. Based on the results from these questionnaires, users were ultimately divided into three categories: users who had not tested positive for the virus and are asymptomatic; users who had not tested positive for the virus but had symptoms such as a fever, cough, or shortness of breath; and users who had tested positive for COVID-19. The collected information from these apps was then locally or remotely processed and then made available to the country’s health care professionals for further analysis.

The fifth most common technical feature was *live statistics and rolling updates*. There were 19 (16.5%) apps that provided live statistics and rolling updates in two forms: Really Simple Syndication (RSS) feeds and push notifications. RSS feeds were used by 14 apps, as shown in Multimedia Appendix 2, to provide up-to-date information about the COVID-19 infection by number of active cases. The cases were then divided into asymptomatic, mild, moderate, severe, recovered, and fatal. These statistics were grouped and illustrated per day, per week, and per month from both a countrywide and worldwide perspective. Additionally, some apps presented the statistics based on hospital admissions within the country rather than active cases. In this same vein, 5 apps used push notifications to provide updates from the government and its advisories, and updated information and subsequent instructions informing of the COVID-19 spread aggregated by the user’s state and country as well as around the world.

The technical feature *latest news* came next in popularity with 16 (14%) apps. A variety of content such as stories, videos, and podcasts were advertised to present the most current global and local news feeds and, in some apps, can be sorted by cities and countries. These apps allow their users to receive immediate COVID-19–related news and updates from trusted nonprofit groups, international organizations, and government agencies in one place.

The technical feature *symptoms tracker* was also supported by 16 (14%) apps. These trackers log symptoms and vitals such as fever and cough at specific frequencies by sending text messages and emails, providing automatic fill-ins for text fields, or sending push notifications to obtain the required data. The collected data from symptom trackers assists in categorizing individuals by severity and health risks into “low-risk” to “high-risk” groups. This method of grouping patients was then used to decide the health care needed for each case. For example, in low-risk symptoms groups, users were provided with knowledge and resources to deal with the disease at home. In higher-risk groups, patients were monitored in anticipation of developing symptoms.

The technical feature *information about health services and care lines* came with 11 apps offering this feature. The apps allowed the users to search nearby hospitals, emergency services, pharmacies, and certified COVID-19 test labs. Some apps consisted of a step-by-step guide on finding testing services and centers that were available around the users’ locations as well as providing the contact information of these services.

The technical feature *map* came with a total of 10 apps. These maps interactively presented both the occurrence of COVID-19 cases (eg, active, confirmed, and recovered cases) as well as the density of these cases in different areas (eg, neighborhoods, cities, and countries). Maps were also used to indicate nearby health care centers and route directions to reach these centers. Moreover, these apps helped health officials observe trends in the community and in turn take meaningful measures to handle the spread of the virus.

The technical feature *health or travel declaration* came with a total of 8 apps. These apps detailed health or travel declarations that were mandated by the country’s government or health officials. Upon arrival, travelers were requested to report themselves and their families through these apps and record their daily health status for 14 days. Moreover, some of these apps were used to notify users of potential exposure risk in the area where the users lived.

The technical feature *location monitoring* came with 7 apps. These apps provided real-time dashboards that help in identifying the next potential COVID-19 hot spots and monitoring and advocating for resources needed in those spots. The collected data were analyzed based on the geolocation of cases and then used to provide support in coordination with local, departmental, and national authorities, which in turn assisted in planning optimal treatment delivery.

The technical feature *sharing data or story with others* was also supported by 7 apps. These apps have the capability to build health diaries describing the users’ symptom development and allow consumers to share their COVID-19 stories with other users and on social networks like Facebook. The stories and diaries can be shared by posting text-based messages, recording voice-based messages, or uploading videos. The users were also able to share these diaries with medical personnel to receive a faster diagnosis.

The technical features *remote monitoring* and *virtual medical consultation* were both offered by 6 apps. Apps that were related to remote monitoring allowed health care professionals to monitor patients’ health data such as heart rate and level of blood oxygen in real time. The generated data could then inform health care teams on how their patients lived on a daily basis and allow them to be immediately alerted if any patient needed critical medical attention. Apps from the virtual medical consultation category enabled virtual medical consultation, live video consultations, or bidirectional text-audio communications to provide personalized support between the users and their doctors.

The technical features *helplines* and *chatbots* were offered by 6 and 5 apps, respectively. Helplines connect users to consultants providing useful information related to COVID-19 and facilitate the introduction of patients to health workers over a toll-free number. Chatbots, on the other hand, are artificial intelligence (AI)–enabled agents who connect with patients through texting or a human-like voice. Chatbots offer personalized health advice via one-on-one conversations with users and help them find answers to their various questions in real time.

The technical feature *distance detection* was enabled by 3 apps. These apps were built to improve the user’s ability to avoid close contact with other people around them. These apps can show how far away the user’s device is from other devices within the same location.

The technical feature *recruitment of volunteers* was offered by 2 apps. These apps were designed to enable the recruitment of volunteers for conducting scientific studies and trials pertaining to COVID-19. These apps asked users to report their symptoms and other required information daily, with each submission generated into data for research purposes.

The technical feature *lists of products* was also supported by 2 apps. These apps present lists of products required for combating COVID-19 (eg, gowns, surgical masks, respirators, face shields, and hand sanitizers), and some allow for calculating the number of these products in stock to find the average consumption rate. This was useful for informing the users about these important products and helping to optimize the use of these resources.

Finally, the least common technical features in this review, each offered in only 1 app, were: checklists of surfaces that required disinfection, taking photos of surfaces, making medical appointments, medical check-up tracking (eg, diagnosis time or submission of diagnosis) during self-quarantine, medical report generators, medication tracking and reminders, mood tracking and mental status (eg, coping with stress), movement permits (eg, during curfew), results of a COVID-19 laboratory test, and using wearable devices for symptom tracking such as pulse oximeters for tracking oxygen saturation in the blood.

**Table 3:** Summary of the apps’ technical features with examples.

Technical features (n=29)	Apps (N=115), n (%)	Selected example of app
Basic health info and advice or FAQs^a^	42 (36.52)	Covid-19 Czechia
Contact tracing	32 (27.83)	TraceCovid
Alert contacts	30 (26.08)	Tabaud
Gadget of self-assessment	20 (17.39)	Covive
Live statistics and rolling updates	19 (16.52)	NCOVI
Latest news	16 (13.91)	CDC^b^
Symptoms tracker	16 (13.91)	Corona Care
Info about health services and care lines	11 (9.57)	CoronApp-Colombia
Map	10 (8.69)	Corona Map
Health or travel declaration	8 (6.96)	Covid-19 Vietnam
Location monitoring	7 (6.08)	Private Kit: Safe Paths
Sharing data or story with others	7 (6.08)	Corona FACTS
Remote monitoring	6 (5.21)	Covidom Patient
Virtual medical consultation	6 (5.21)	Laziodr Covid
Helpline	6 (5.50)	Covid-19 UAE
Chatbot	5 (4.35)	HealthLynked COVID-19
Distance detection	3 (2.61)	VírusRadar
Recruitment of volunteers	2 (1.74)	Covid Radar
List of products for combating COVID-19	2 (1.74)	NIOSH^c^ PPE^d^ Tracker
Checklist of disinfected surfaces	1 (0.87)	Disinfection Checklist
Making medical appointments	1 (0.87)	GVA CoronVirus
Medical check-up tracking	1 (0.87)	Self-Quarantine app
Medical report generator	1 (0.87)	Premedicus
Medication tracking and reminders	1 (0.87)	Patientsphere for Covid19
Mood tracking and mental status	1 (0.87)	Covid Coach
Movement permits	1 (0.87)	Tawakkalna KSA^e^
Results of COVID-19 laboratory test	1 (0.87)	Tatamman
Taking photos of surfaces	1 (0.87)	Disinfection Checklist
Wearable devices for symptom tracking	1 (0.87)	PatientMpower for COVID-19

^a^FAQ: frequently asked question.

^b^CDC: Centers for Disease Control and Prevention.

^c^NIOSH: National Institute for Occupational Safety and Health.

^d^PPE: personal protective equipment.

^e^KSA: Kingdom of Saudi Arabia.

### Purposes of Apps Related to COVID-19

**Figure 3:**
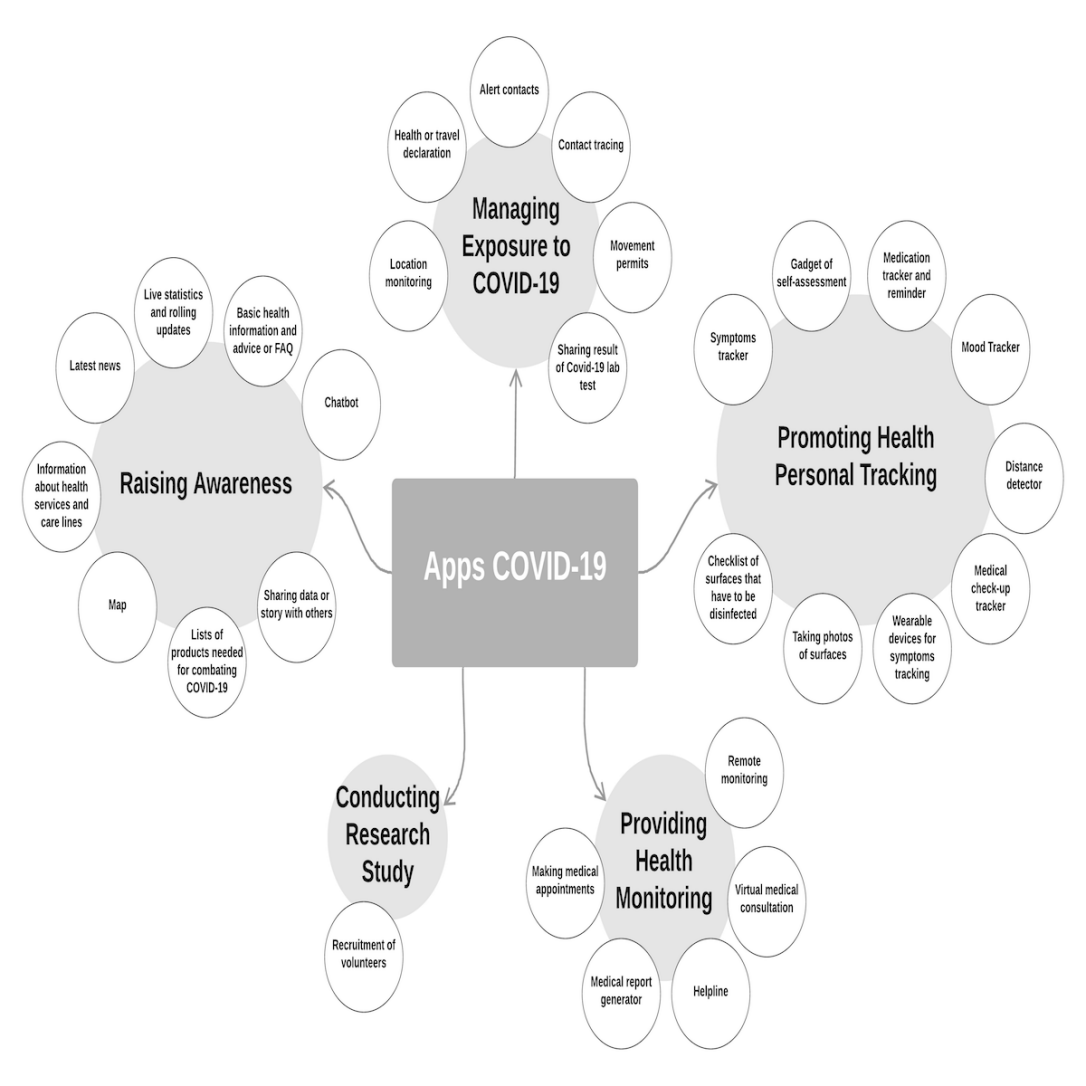
Taxonomy of COVID-19 apps’ purposes. FAQ: frequently asked questions.

The identified technical features (n=29) were then analyzed and organized into five dimensions that represented purposes of these health apps. This led to the development of our taxonomy for health apps related to COVID-19, as shown in Figure 3. These purposes were tracking personal health, raising awareness, monitoring health by health care professionals, managing exposure to COVID-19, and conducting research studies. The majority of technical features were related to the first two purposes. Each purpose is described in the following section.

For tracking personal health, a total of 9 (31%) technical features out of 29 were found relevant, as the data generated from these apps helped their users look after their health. These technical features were gadgets of self-assessment used as an initial triage of possible infection, symptom trackers to check oneself for COVID-19 symptoms on a daily basis, medication trackers and reminders, mood trackers, distance detectors to maintain a safe distance from others, diagnosis recorders used during self-quarantine, integrated cameras for taking photos of surfaces that have been disinfected, checklists of surfaces that have to be disinfected for tracking hygiene practices, and wearable devices for tracking symptoms.

For raising awareness about COVID-19, 8 (28%) technical features out of 29 were categorized under this dimension, as these apps were concerned with providing various data and information to help users stay informed about this disease. These technical features included providing basic health information and advice or FAQs, presenting live statistics and rolling updates, showing the latest news, offering information about health services and care lines, providing interactive maps of active cases and nearby medical facilities to help users learn about this information, chatbots to answer the user’s questions, incorporating lists of products needed for combating COVID-19 to learn about this important precaution, and allowing users to share data and stories with others such as family members and doctors to inform them about their health status and the most current health care information and practice.

For managing exposure to COVID-19, 6 (21%) technical features were classified under this dimension, as these apps help users avoid being exposed to this virus. These features were contact tracing, alerting users who were within close proximity of someone who had tested positive for the virus, reporting suspected cases and declaring travel after arrival, granting movement permits during curfews, monitoring the location of consenting users to further understand trends of COVID-19 within various communities, and tools for sharing results of certified COVID-19 examinations with users.

For health monitoring, 5 (17%) technical features were placed under this dimension, as these apps help users to seek medical assistance from their health care professionals. These features were remote monitoring by health care professionals, virtual medical consultations with clinicians via video or audio calls, medical report generators, tools for making appointments to visit health centers, and helplines to obtain necessary medical help.

Finally, for conducting research studies, many features such as symptoms tracker, distance detector, and self-assessment gadgets enabled this dimension, but only 1 (3%) technical feature uniquely supported it. This feature was intended to enable researchers to recruit volunteers to take part in COVID-19–related studies and clinical research across countries.

## Discussion

### Principal Findings and Comparison to Prior Studies

This app review shows that the health apps related to COVID-19 vary in terms of their developer type, basic technical features, and purposes of use to combat COVID-19. Regarding the developer types, this review reveals the high number of apps developed by governments or national authorities to fight this infectious disease. In comparison, apps related to noncommunicable diseases like diabetes and hypertension are mostly developed by nongovernmental entities such as health care providers and hospitals [19,20]. This indicates the essential role that apps could play as powerful “weapons” in public health crisis management [32].

The most common technical features in this review focused on offering basic health information and advice on COVID-19 followed by contact tracing. However, the authors noticed that over the review period the number of health apps related to contact tracing was increasing. For example, health apps released at the early stages of the COVID-19 pandemic (ie, February, March, April, and May 2020) tended to be concerned with raising awareness more than other purposes. Apps released in June, July, August, and early September 2020 tended to offer COVID-19 exposure notifications more than any other purpose (see Multimedia Appendix 1). Moreover, it is also expected that contact tracing apps will continue to increase [33,34]. As COVID-19 is highly contagious, digital contact tracing is preferred by many health agencies and governments to expand their capacity for fighting the rapid spread of the virus [34,35]. Contact tracing via apps is also deemed more effective compared to traditional data collection [36]. In traditional contact tracing, tracing is performed through hired tracers and notifications are made by phone calls, which is costly and time consuming, and the chance of missing potential exposures is high [36].

This review also observed the increase in AI-based technical features for providing customized support to fight against the COVID-19 outbreak. For example, AI-enabled health education agents are offered through chatbots. Although 4 apps included in this review had chatbots within their technical features, the number of chatbot apps and their popularity are expected to increase [37]. Health chatbots can provide knowledge about the symptoms caused by the novel SARS-CoV-2 virus, teach their users how to detect if they have been affected by or infected with the virus, and present them with recommendations based on CDC guidelines and advice to reduce the risk of infection [10,38].

Moreover, some researchers are concerned about future lasting effects of the virus, which can be somewhat mitigated with currently available app technology [39]. For instance, Neubeck et al [40] conducted a review of the effectiveness of remote care to cardiovascular patients during the era of COVID-19. They found that there were two specific limitations that could not yet be answered by current mobile apps: rapidly changing symptoms and social isolation. This review found similar limitations in the current technology. Although chatbots are excellent in the initial stages of assessing symptoms or answering questions, they are not advanced enough to provide a sense of human contact that isolated or immunocompromised users may be interested in.

Regarding the purposes of apps, tracking personal health and raising awareness were dominant in this review and in other relevant reviews of COVID-19 apps [41,42]. As the COVID-19 outbreak has progressed, monitoring peoples’ health and adherence to prevention procedures—including mobility, early detection of mild symptoms, and providing necessary psychological support—by health officials and national health care providers has become increasingly difficult to maintain and is anticipated to become even more challenging with an ever-increasing number of cases [43,44]. To combat these likely upcoming issues and at the request of health officials, many of the apps identified in this review had personal health-tracking capabilities. These apps support public health officials’ emphasis on the importance of personal health tracking as a participatory approach to curtail further spread of COVID-19 in the community [45]. By involving most of the population in self-tracking their personal health and providing them with the technology to self-assess, the population can work in tandem with health care providers to combat the effects of the virus.

Additionally, in the 51 countries that are presented in this review, informing the community on how to mitigate their exposure to COVID-19 has been provided by digital health technologies [2]. The authorities in these countries and other stakeholders (eg, health care providers and policy makers) have used apps that provide services such as contact tracing and widespread alerts. Additionally, these apps assist in restricting travel and keeping contact with users, which shows the imperative need for these apps to keep the community safe. Likewise, as health care providers can use these apps to stay informed on the community’s needs, they can in turn plan on how to best deliver treatment services. Each of these groups is responsible for their duty in combatting the virus; in using this technology, various stakeholders can feel confident in knowing what to do for reducing the risk of COVID-19 [2,6].

### Implications of Findings

This study provides a holistic review of the technical features that are common in health apps related to COVID-19 and identifies the most used ones for various purposes as shown in Figure 3. Knowledge of both the technical features and their applications in combating COVID-19 may help developers in making informed decisions when designing apps for various stakeholders.

For governments and health officials, our review shows not only that health apps can support several different methods in which to mitigate the effects of COVID-19 but also that its information can be accessed rapidly and inexpensively [6]. This review shows the ability of COVID-19–related apps to quickly and easily transmit data to both public health centers and treatment providers as well as epidemiologists, virologists, and clinicians. These health apps are equipped with different technical features such as contact tracing, where information can be uploaded and transmitted, privately held and maintained by health care authorities, yet still accessible to the public [46]. Individuals can use these apps to provide information about themselves or their social circle and can be alerted when approaching an area where the risk of exposure is high. These insights may encourage governments and health officials to request building apps in a way that can expand consumers’ capabilities to feel educated, protected, and informed of their own health information and their community during pandemics.

For researchers in public health and medical informatics, there is enormous potential for future research and app-based development with regard to how mobile apps have so far been used to combat COVID-19, as shown in this review, and will ideally be used in the future [47]. One of the most frequent questions about these apps is related to their acceptability [48] and effectiveness [49]. These assessments, which are based on the apps’ technical features (eg, the medication reminders, information on COVID-19 that the users can take advantage of), can inform not only successful individual adoption of these technologies but also effectiveness of overall services that apps can provide [47,50]. Our taxonomy of health apps purposes, illustrated in Figure 3, can be used as a guidance tool in categorizing apps and, assessing their functionalities, and evaluating their acceptability and effectiveness in each category.

Lastly, for average consumers, as health apps related to COVID-19 are low-cost and publicly available resources, this study provides a holistic reference of apps that are currently available in the market across 51 countries. Multimedia Appendix 1 is rich with information about the health apps’ specifications and includes their webpages for convenient access. Multimedia Appendix 1 may help to educate consumers about various apps available and their different functionalities, which may improve their abilities to choose the best suitable app based on their needs and requirements [42].

### Recommendations for Future Studies

As countries who are actively collecting data are better equipped to make decisions on how to best combat COVID-19, it is recommended to investigate and find approaches for encouraging as many people as possible to use these apps [17,50,51]. With an app, a user can at any time conduct a self-assessment to evaluate any symptoms they may have and take appropriate measures to keep themselves and their community safe. However, poor choices made by developers and governments about the apps’ designs have led to technical flaws and security concerns that could make the apps less powerful and may hinder consumers’ willingness to use them [17]. Therefore, these types of apps should be developed with health informatics experts to improve collaboration between government, health care organizations, and app developers, and to achieve the best quality of data collection and protection [32].

Self-monitoring–related apps have played a large role in health care provision, treating patients without being overwhelmed by in-person visits and enabling treatment providers and patients to use symptom progression tracking in real time [11]. However, health monitoring has to be distinguished as a concept from health tracking [52]. In health monitoring, the health care professionals, not users, take the initiative and provide guidance for their patients through the treatment course. In health tracking, the user takes initiative to complete actionable steps for health self-management [52]. Therefore, it is recommended that this difference be taken into consideration when the evaluation of app functionalities and applications is performed.

Additionally, although daily or even more frequent reminders of medication adherence, filling out self-assessments, and recommendations to self-quarantine may be effective for some users, others need more specialty care [53]. People who are at risk for rapidly changing symptoms must be informed that, when a new symptom suddenly develops, it is imperative that they immediately seek in-person treatment. Most of the apps included in this review did not explicitly have this type of technical feature. Although it has been important that these initially developed apps can answer the questions of a universal audience, there is a need for apps to be nationally accessible but exceptionally customizable.

Furthermore, one of the most pressing questions in clinical treatment and research is how to prevent the feelings of social isolation that quickly developed in a large portion of the population—particularly in older adult and immunocompromised populations [54]. There has been little research on the use of mobile apps to combat symptoms such as loneliness in a worldwide lockdown like the one enacted for COVID-19 [51]. Considering the individual and social effects of the virus that apps are not yet answering and in anticipation of future public health emergencies, medical experts and app developers looking to create innovative and useful products may want to consider amplifying a more personalized experience with more opportunity for human interaction. Future research can be conducted on both how feelings of depression, isolation, and loneliness may be reduced with the use of mobile health apps and if a personalized experience leads to beneficial, cost-effective in-person treatment [44].

Lastly, few health apps were used for conducting research studies. In this review, this was found to be the overall least common purpose of health apps related to COVID-19. Obtaining informative data on the novel SARS-CoV-2 virus from consumers is essential for public health specialists and medical researchers to successfully carry out their studies, which will inform future understanding of the infection and risk factors for adverse outcomes, characterize the virus transmission patterns, identify high-risk patients, and eventually assist clinicians in fighting COVID-19 [43,55]. Therefore, further effort is required from governmental organizations to promote the conduct of participatory disease-based studies of the COVID-19 pandemic through the development of these digital solutions [45].

### Limitations of This Study

This review has its own limitations. Our search for health apps was limited to the major app stores Apple Store and Google Play. However, these stores are the largest global platforms for app distribution, with 4.41 million apps as of May 2020, which accounted for about 80% of apps in the market [56]. Additionally, as searching all national app stores from a single country is difficult since international apps are not visible, other apps may have been missed [41,42]. Thus, other sources (eg, news and the Google search engine) were used to search for apps to overcome this problem. Furthermore, the quality of these apps was not examined and rated as suggested by Stoyanov et al [57], but rather, the apps’ information that was retrieved from the app stores was thoroughly described and presented in Multimedia Appendixes 1 and 2. In addition, as 67% (77/115) of the included apps were developed by national health officials or governments, accessing some of these apps’ content required national identification codes or local country phone numbers; this led the authors to making the decision to not perform an assessment of these apps’ quality, as most information would be inaccessible [36,42].

### Conclusions

This study provides an overview and taxonomy of the health apps currently available in the market to combat COVID-19 based on their differences in terms of basic technical features and purposes of use. The analysis of 115 health apps related to COVID-19 led to extracting 258 excerpts that were grouped into 29 technical features as shown in Table 3. These technical features were then categorized, which led to five overarching dimensions or purposes of apps for fighting COVID-19 as shown in Figure 3. Further effort is required from researchers to evaluate these apps’ effectiveness and acceptability, and from governmental organizations to increase public awareness of these digital solutions. Our taxonomy of these apps’ purposes can be used as a guidance tool in categorizing apps and then assessing their functionalities, as well as to evaluate their effectiveness in each category.

## Appendix

Multimedia Appendix 1 Summary of the apps included in the review.

Multimedia Appendix 2 The occurrence of excerpts or technical features in each app.

## Abbreviations

AI: artificial intelligence
CDC: Centers for Disease Control and Prevention
FAQ: frequently asked questions
PRISMA-ScR: Preferred Reporting Items for Systematic Reviews and Meta-Analyses Extension for Scoping Reviews
RSS: Really Simple Syndication
SARS: severe acute respiratory syndrome
STI: Secure Tracing Identifier
WHO: World Health Organization
