# Supplementary material for: Health Apps for Combating COVID-19: Descriptive Review and Taxonomy
Source: JMIR Mhealth Uhealth. 2021 Mar 2;9(3):e24322. doi: 10.2196/24322 (PMC7927949; doi:10.2196/24322)
Supplement: Multimedia Appendix 1 [file mhealth_v9i3e24322_app1.pdf]

Appendix 1

	Name	Version Date	Country of origin	Technical Features	Author/Developer	Source
1.	HealthLynked COVID-19 Tracker	26 Feb 2020	US	• CoronaVirus tracker (Users can update their Information as symptoms change and these changes are reflected on the Map).<br>• Map that shows the latest news about the virus from around the world to keep users updated on the Virus (Data sources: DXY, Worldometer, Johns Hopkins).<br>• Alert contacts (if they test positive for the virus).<br>• The lasted WHO data indicated by the following Criteria: WHO confirmed Infections are shown in RED, WHO deaths are shown in BLACK.<br>• User can self-report and they are divided into three categories: Users that have NOT tested positive for the virus and are asymptomatic are shown as DARK GREEN; users that have NOT tested positive for the virus but have symptoms such as a fever, cough or shortness of breath are shown as YELLOW; users who self-report that they have tested positive for the CoronaVirus are shown as PINK.<br>• Users can also engage in real-time chat with the chat feature.	Healthlynked Corp.	https://apps.apple.com/us/app/healthlynked-covid-19-tracker/id1500575377
2.	Coronavirus-SUS	28 Feb 2020	Brazil	• Information on various topics such as symptoms. how to prevent, what to do in case of suspicion and infection and etc.<br>• Map indicating nearby health units. In case of suspected infection, the citizen can check if the symptoms are compatible with Corona's, and if so, will be instructed and referred to the nearest basic health unit.<br>• Official news area of the Ministry of Health focusing on CoronaVirus. (Translated)	Governo Do Brasil	https://apps.apple.com/sa/app/coronav%C3%ADrus-sus/id1408008382
3.	Relief Central Covid-19	3 Mar 2020	US	• Coronavirus (COVID-19) Outbreak Guidelines, an exclusive resource developed to help the health care community stay up-to-date. It includes the latest versions of The World Factbook from the CIA, CDC Health Information for International Travel. Guide from USAID delivers disaster assessment and response information and includes chapters and appendices ranging from information on populations at risk to commonly used acronyms, all arranged in an outline format.<br>• Relief News puts the latest news at your fingertips with a collection of RSS feeds from trusted non-profit groups, international organizations, and government agencies.	Unbound Medicine, Inc.	https://apps.apple.com/sa/app/relief-central-covid-19/id353219185
4.	Covid-19 Vietnam	6 Mar 2020	Vietnam	• Chatbot (Virtual Medical Assistant): conducts one-on-one conversations with users by human sounding voice, works in real-time and answers the questions about COVID-19 on the spot.<br>• Board-certified specialist consultation: allow people to interact with a group of specialists at National Hospital for Tropical Diseases by chatting, audio/video calling for medical advices.<br>• Interactions between government and citizens: Users can send comments, requests, supports, and Health or travel declaration.<br>• Users can get the most recent advices and notices from the Government, WHO, CDC, NHC, Johns Hopkins University and other medical institutions. Prevention of COVID-19: provides guidance documents, videos, animation clips from WHO, Vietnam Ministry of Health and other medical institutions.<br>• COVID-19 Live Updates: shows live statistics and rolling updates from coronavirus disease in Vietnam and the world. The numbers of cases per day, per week, per month, confirmed cases, increased numbers, etc. are analyzed and visually shown in map, graphics, number tables with comparisons and simulations by a group of data analysts continuously working 24/7.<br>• COVID-19 Map: automatically synchronizes world wide data from WHO, CDC, NHC, etc.<br>• Enables users to search nearby hospitals, pharmacies, and certified COVID-19 test labs.	Ministry of Health: Advanced International Joint Stock Company (AIC Group) & Electronic Health Administration	https://apps.apple.com/sa/app/covid-19/id1501810040
5.	Covidom Patient	10 Mar 2020	France	• Remote monitoring, the healthcare team knows how the patient lives on a daily basis and is alerted if the patient needs more specific attention. (Translated)	Assisstance Publique-Hopitaux De Paris	https://apps.apple.com/sa/app/covidom-patient/id1501889139
6.	NCOVI	11 Mar 2020	Vietnam	• Declare health information of themselves and their families anytime, anywhere. Send information on the disease COVID-19, the suspect object around the area where he/she lives.	Authority of Information Technology Application	https://apps.apple.com/sa/app/ncovi/id1501934178

				● Update statistics on COVID disease information 19 quickly and officially from the Ministry of Health.<br>● Receive recommendations and instructions to effectively prevent diseases and reduce the risk of nCov infection. Get comprehensive information about the disease to make appropriate decisions. (Translated)		
7.	Bolivia Segura	13 Mar 2020	Bolivia	● Online self-assessment<br>● Active monitoring (GPS)<br>● Daily record of symptoms<br>● Frequently asked questions<br>● Official Data<br>● Latest News (Translated)	Agencia De Gobierno Electronico Y Tecnologias De Informacion	https://apps.apple.com/sa/app/bolivia-segura/id1502299114
8.	PatientMpower for COVID-19	14 Mar 2020	Republic of Ireland	● Monitor your oxygen saturation (using a device called a pulse oximeter), which is important to track to ensure you are recovering from COVID-19. Tracks your symptoms including breathlessness. Records details of relevant underlying conditions and your existing medications.<br>● Anonymised location monitoring to help us understand trends of COVID-19 across Ireland and plan resources to best deliver healthcare.<br>● Syncs with Apple Healthkit to track heart rate and activity Confidential health data viewable in real time by healthcare professionals at monitoring centres.	Patientmpower LTD	https://apps.apple.com/sa/app/patientmpower-for-covid-19/id1502617117
9.	Nua health Video Consultations	16 Mar 2020	Republic of Ireland	● Live Video Consultations – medical advice from your own clinician using video and audio.	Webdoctor Limited	https://apps.apple.com/sa/app/nuahealth-video-consultations/id1502827324
10.	COVID-19 Medisch Dossier	17 Mar 2020	Netherlands	● Daily developments around Corona, and knowledge to take the measures to prevent infection of others and yourself.<br>● Show "hospital admissions" instead of "active cases" according to new RIVM information<br>● News section and Push Notifications (translated).	Medischdossier.Org	https://apps.apple.com/qa/app/covid-19-medisch-dossier/id1502322865
11.	Stop Covid19	18 Mar 2020	Spain	● Questionnaire that indicates whether they have the possibility of having COVID.<br>● Collect population data in order to be able to create heat maps for the dashboard. (Translated)	Generalitat De Catalunya	https://apps.apple.com/sa/app/stop-covid19-cat/id1502992288
12.	GoK Direct	18 Mar 2020	India	● A user can find information from government services in his/her area by searching the area's zip code<br>● A user can receive COVID19 alerts and official news updates from the government.	Government of Kerala	https://apps.apple.com/sa/app/gok-direct/id1502436125
13.	TraceTogether	18 Mar 2020	Singapore	● The app uses Bluetooth, and your Bluetooth data is stored securely on your phone. It'll only be shared with MOH if you test positive for COVID-19, for the sole purpose of contact tracing.<br>● Notifies you quickly if you've been exposed to COVID-19 through close contact with other TraceTogether users.<br>● Allows the MOH to give you timely care and guidance, protecting you and those around you.	Government Digital Services agency of Government Technology Agency of Singapore	https://apps.apple.com/sa/app/tracetogether/id1498276074
14.	Laziodr Covid	19 Mar 2020	Italy	● The Lazio Region offers the Lazio Doctor platform to consult useful information related to the COVID-19 emergency and to connect patients with health workers and toll-free number 800118800.<br>● Self-assessment questionnaire, some information will be processed by the system and will be made available to healthcare professionals.<br>● Provide secure bidirectional text-audio communications via smartphone between the citizen and their doctor. (Translated)	Lazio Crea	https://apps.apple.com/sa/app/laziodrcovid/id1503189116
15.	Osler Covid Learning Centre	24 Mar 2020	US	● This app is designed to support nurses, doctors, paramedics and allied health professionals to learn and refresh their skills and knowledge, helping them perform these expanded roles. The full catalogue of modules is fully searchable and is available to all app users. It also provides access to a series of podcasts related to the COVID-19 pandemic, along with a regularly refreshed resources page.	Osler Technology Pty Ltd	https://apps.apple.com/sa/app/osler-covid-learning-centre/id1504015523
16.	Patientsphere for Covid19	20 Mar 2020	US	● Log important health data such as fever, cough<br>● Medication tracking and reminders.<br>● Share your symptoms with family; and doctors and help them to accurately diagnose the disease.	Open Cancer Network	https://apps.apple.com/sa/app/patientsphere-for-covid19/id1445938461

17.	Coronavirus UY	20 Mar 2020	Uruguay	• Perform an analysis for the Coronavirus COVID-19, and follow up on your case and the next steps you will need to take. (translated)	Agesic	https://apps.apple.com/sa/app/coronavirus-uy/id1503026854
18.	CoronApp-Colombia	20 Mar 2020	Columbia	• Report symptoms and those of family members.<br>• Receive recommendations according to state of health after making the self-diagnosis.<br>• Access official information on the Cases of Coronavirus in Colombia.<br>• Obtain information about health services and care lines.<br>• Facilitate real-time monitoring of the data collected so that the INS Emergency Operations Center can act quickly and provide support in coordination with local, departmental and national authorities. (Translated)	The Colombian Government and National Institute of Health	https://apps.apple.com/sa/app/coronapp-colombia/id1502037648
19.	BC Covid-19	22 Mar 2020	Canada	• Stay informed about COVID-19 in BC and determine what actions and next steps you should take and the latest BC guidelines related to COVID-19.<br>• Receive timely updates with important news and alerts from BC's Ministry of Health.	Her Majesty the Queen in Right of The Province of British Columbia	https://apps.apple.com/sa/app/bc-covid-19-support/id1502907052
20.	BMC Combat COVID-19	25 Mar 2020	Mumbai, India	• Help track your health progress during self- quarantine, and contain the spread of this virus.	Municipal Corporation of Greater Mumbai	https://apps.apple.com/ar/app/bmc-combat-covid19/id1504083673
21.	Family-Covid19	26 Mar 2020	Vietnam	• Self-declaration of disease symptoms and epidemiological factors for patients, relatives and partners at Family Hospital (Da Nang) to protect the health of individuals and the community against complicated developments of the epidemic COVID-19. (Translated)	Family Healthcare Joint Stock Company	https://apps.apple.com/sa/app/family-covid-19/id1504207443
22.	SOS CORONAVIRUS	26 Mar 2020	Mali	• Inform and raise awareness of the dangers of covid-19 and ensure better management of suspect cases. (Translated)	Agetic Mali	https://apps.apple.com/sa/app/sos-coronavirus/id1504728171
23.	Covid-19 Czechia	27 Mar 2020	Czechia	• Provide basic information about the infection, how to prevent it and how to defend against it.<br>• Up-to-date information about the infection in numbers, both worldwide and within the selected country.<br>• The latest information and news.<br>• Map of the occurrence of COVID-19 and within the map you can display information about a specific country throughout the world.	Nemocnice Milosrdnych Bratri P.O. (Merciful Brothers Hospital)	https://apps.apple.com/sa/app/covid-19/id1504906590
24.	Trecovid19	27 Mar 2020	Italy	• Do you have doubts or questions about the Coronavirus emergency? Do you want to know what measures are foreseen in Trentino? What are the numbers to contact in case of need?	Azienda Provincial Per I Servizi Sanitari – Provincial Autonoma Di Trento	https://apps.apple.com/sa/app/trecovid19/id1503151087
25.	Stopp Corona	27 Mar 2020	Austria	• keep track of encounters with friends, family or co-workers.<br>• Should you contract the corona virus all your encounters of the last two days will be informed automatically and anonymously.<br>• Helping all of you to take appropriate measures such as keep a safe distance (1–2 meters) from others, avoid social contact, self-quarantine as a precaution.<br>• How are you feeling today? Just answer the clinically proven questionnaire to check yourself for corona symptoms daily.<br>• Report suspected infections.	Austrian Red Cross	https://apps.apple.com/sa/app/stopp-corona/id1503717224
26.	Corona FACTS	28 Mar 2020	US	• Transmits global news feeds that can be sorted by country, so you can track news in one place<br>• Case Maps: Displays interactive maps, including county-specific data for the U.S., with case details that are updated daily<br>• Evidence-Based Content: Provides FAQs and other information curated by clinicians, with relevant citations of the source so you can trace the foundation of the answers<br>• My Story: Upload videos to share your COVID-19 story, note of thanks, or words of encouragement to others around the globe (share on Facebook as well). Library: Submit your own articles for inclusion on the site	Trusted Medical Llc	https://apps.apple.com/sa/app/coronafacts/id1504490714

27.	Coronavirus Australia	28 Mar 2020	Australia	● The official information and health advice to help stop the spread and stay healthy get a quick snapshot of the current official status within Australia<br>● Check symptoms if you are concerned about yourself or someone else find relevant contact information<br>● Access updated information from the Australian Government<br>● Push notifications of urgent information and updates	Digital Transformation Agency	https://apps.apple.com/sa/app/coronavirus-australia/id1503846231
28.	MySejahtera	28 Mar 2020	Malaysia	• It allows users to perform health self-assessment on themselves and their family members.<br>• The users can also monitor their health progress throughout the COVID-19 outbreak.<br>• It enables the Ministry of Health (MOH) to monitor users' health condition and take immediate actions in providing the treatments required.	National Security Council / Ministry of Health	https://apps.apple.com/sa/app/mysejahtera/id1504055630
29.	BeAware Bahrain	28 Mar 2020	Bahrain	• It assists efforts to contain the spread of COVID-19 by advancing contact tracing efforts.<br>• The app utilizes location data shared by users to alert the people in close proximity with an active case.	eGovernment Authority Bahrain	https://apps.apple.com/sa/app/beaware-bahrain/id1501478858
30.	Canada Covid-19	30 Mar 2020	Canada	● Recommendations are personalized and based on your personal risk factors.<br>● News and alerts from Canada's Ministry of Health.	Thrive Health	https://apps.apple.com/sa/app/canada-covid-19/id1505010304
31.	COVID-19 TAM	30 Mar 2020	Tamaulipas State, Mexico	● Provides information on the total number of cases under investigation, as well as the cases that have been discarded, active cases (divided into asymptomatic, mild, moderate and severe), recovered and fatal.<br>● allows geolocation of cases, in order to help prevent their spread and establish proper follow-up on them. (Translated)	Gobierno Del Estado Tamaulipas	https://apps.apple.com/ar/app/covid-19-tam/id1505380068
32.	Covid-19 UAE	1 Apr 2020	UAE	● Real-time Coronavirus cases information and regional news & events.<br>● For patients, app offers a full stack of services ranging from Live Calls, Quarantine management and support.	Ministry of Health	https://apps.apple.com/sa/app/covid-19-uae/id1504980550
33.	Corona Care	1 Apr 2020	US	● Track COVID-19 symptoms of potentially infected patients that have been examined at a clinic or ER and need to be sent home, and helps healthcare providers in their research and see if individuals have moved from low to high-risk groups. enables providers to categorize patient cases by severity and mitigate risks of public spread by providing all patients with knowledge and resources to deal with low-risk symptoms at home, while also tracking the development of symptoms in higher-risk patients. Surveys can be sent to targeted groups at specific frequencies via push notification, email, or text.<br>● Provide instructions to patients on the app-Bidirectional instant message and video calls between providers and patients.<br>● Al - enabled chatbot to help patients with questions more effectively.	Paxera Health Corp	https://apps.apple.com/sa/app/corona-care/id1503322438
34.	AarogyaSetu	2 Apr 2020	India	● It is aimed at augmenting the initiatives of the Government of India in proactively reaching out to and informing the users of the app regarding risks, best practices and relevant advisories pertaining to the containment of COVID-19. (translated)	National Informatics Centre	https://apps.apple.com/sa/app/aarogyasetu/id1505825357
35.	Covid-19 Armenia	2 Apr 2020	Armenia	● The latest official news about COVID-19 in Armenia.<br>● You can determine if COVID-19 signs are present and understand whether or not you should visit a medical facility.	Office of The Prime Minister of The Republic of Armenia	https://apps.apple.com/sa/app/covid-19-armenia/id1505830061
36.	Castor covid-19	2 Apr 2020	US	● Recruitment of healthy volunteers to support COVID-19 studies and clinical research across the globe.<br>● This app asks healthy volunteers to report their symptoms on a day to day basis.<br>● Population can be monitored by trained healthcare professionals through real-time dashboards.	Castor	https://apps.apple.com/sa/app/castor-covid-19/id1504270201
37.	Covid Radar	2 Apr 2020	Netherlands	● We want to be able to better predict what care needs will soon be in the Netherlands: how many corona patients do we expect where and when? We only test someone if he / she has symptoms and belongs to a vulnerable target group. (Translated)	Academisch Ziekenhuis Leiden	https://apps.apple.com/sa/app/covid-radar/id1504947356
38.	Premedicus (SAR 74.99)	2 Apr 2020	US	● It asks you a few questions in everyday language and give you personalized advice about what to do next. Diagnosis is much more accurate because you provide background information about your health, and it is hospital tested.<br>● Triage platform	PreMedicus LLC	https://apps.apple.com/sa/app/premedicus-er/id1505306194

				• COVID-19 checkup & advice is included that allow you to learn more about the possible condition.<br>• Provide GPS guidance to the right type of care provider if you decide to seek help from a doctor.<br>• Create an illness Report using medical terms that you can email to your doctor.		
39.	Rakning C-19	2 Apr 2020	Iceland	• The app collects the GPS location of the phone and stores locally on the device. If the phone owner is diagnosed with the Covid-19 disease he is asked by the Directorate of Health to share the location data for contact tracing in order to identify individuals that might need to go into quarantine.	Landlaeknisembaettid	https://apps.apple.com/ar/app/rakning-c-19/id1504655876
40.	Aarogya Setu	2 Apr 2020	India	• Informing the users of the app regarding risks, best practices and relevant advisories pertaining to the containment of COVID-19.	Union Government of India/National Informatics Centre	https://apps.apple.com/in/app/aarogyasetu/id1505825357
41.	How We Feel	2 Apr 2020	US	• Self-report your symptoms daily, even if you feel healthy. It only takes a minute.<br>• See how many people symptoms near you have, to keep you and your neighbors safe.<br>• Get updates about efforts to fight the outbreak and tips to help you stay healthy.	The how we feel project, Inc	https://apps.apple.com/sa/app/howwefeel/id1503942026
42.	TraceCovid	3 Apr 2020	UAE	• It allows users to detect another device with the same installed app and share proximity data with it. For example, when you are at a supermarket, upon close proximity with someone who has also installed the TraceCovid app as you do, the app on both your devices will exchange an encrypted Secure Tracing Identifier (STI) and store the exchanged STI locally on your devices. The STI consists of anonymised data and timestamp. No personally identifiable information is collected. When one of the users is infected with the virus, the official authorities will request the infected user to upload the list of STI stored locally on his or her device.	Department of Health Abu-Dhabi	https://apps.apple.com/sg/app/tracecovid/id1505485835
43.	Disinfection Checklist	3 Apr 2020	US	• The app contains a comprehensive checklist with 38 checks to ensure that all surfaces have been disinfected.<br>• The app collects photos, signatures and dates, and submits the inspection report into a convenient PDF file that can be shared with your clients via email or other means available on your device.<br>• The app also contains a list of products that have been pre-approved by the U.S. Environmental Protection Agency (EPA) for use against emerging enveloped viral pathogens and can be used during the 2019 novel coronavirus (COVID-19) outbreak.	Snappii Corp.	https://apps.apple.com/sa/app/disinfection-checklist/id1504450491
44.	Asistencia COVID-19	6 Apr 2020	Spain	• Self-assess the possible symptoms of COVID-19 infectious disease and find out about the recommendations to follow, allowing an initial triage of possible cases.<br>• Subsequent follow-up by the Health Authorities. (Translated)	Ministerio De Asuntos Economic Y Transformacion Digital	https://apps.apple.com/sa/app/asistencia-covid-19/id1504750846
45.	MorChana	6 Apr 2020	Thailand	• It will track infection, probability and risk of COVID-19 and observe the outbreak situation from data provided by you.	Electronic Government Agency	https://apps.apple.com/sa/app/morchana-%E0%B8%AB%E0%B8%A1%E0%B8%AD%E0%B8%8A%E0%B8%99%E0%B8%B0/id1505185420
46.	GVA CoronVirus	8 Apr 2020	Spain	• Request an appointment with your health center in case of presenting clinical symptoms compatible with COVID-19 infection, and you will be informed of the status of the appointment: requested, confirmed, made.<br>• Extensive information about the infection caused by the COVID-19 coronavirus can also be accessed through the APP. (translated)	Generalitat Valenciana	https://apps.apple.com/sa/app/gva-coronavirus/id1504239771
47.	Covid19-dxb Smart App	10 Apr 2020	UAE	• Information about COVID-19 Symptoms, how you can prevent yourself from it and what treatments can be adopted to reduce the risk.<br>• Real statistics worldwide and within the United Arab Emirates. This is helping to stop spreading fake stats, counter fake NEWS circulating around people and gives<br>• Personalized support from designated paramedics in case you are infected by COVID-19.	Dubai Health Authority	https://apps.apple.com/sa/app/covid19-dxb-smart-app/id1504818399

48.	Covid Watcher	10 Apr 2020	US	• It will help identify the next COVID-19 hot spots or which resources are needed in the neighbourhood.<br>• Your answers contribute to insights we return through online.<br>• Interactive maps that visualize in an anonymous and aggregated way the pandemic's evolution in different neighbourhoods.	Columbia University	https://apps.apple.com/sa/app/covidwatcher/id1504295590
49.	COVID Puebla	10 Apr 2020	State of Puebla, Mexico	• Offers you an immediate, reliable and official self-evaluation. (Translated)	Secretar A De Finanzas Y Administraci N Del Gobierno Del Estado De Puepla	https://apps.apple.com/sa/app/covid-puebla/id1507242578
50.	Shlonik	10 Apr 2020	Kuwait	• It provides the latest health updates<br>• a health Bot<br>• a self-check-in mechanism for quarantined patients<br>• vitals reporting<br>• a communication tool with the MOH medical teams.	The Central Agency for Information Technology	https://apps.apple.com/sa/app/shlonik-%D8%B4%D9%84%D9%88%D9%86%D9%83/id1503978984
51.	Covive	11 Apr 2020	Global	• Evaluate the probability of having contracted COVID-19<br>• Monitor your symptoms and vitals whether you have tested positive for the virus or not.<br>• Up-to-date information to protect yourself and your community	BioneXt Lab	https://apps.apple.com/sa/app/covive-your-covid-19-app/id1507215956
52.	Stop Covid	12 Apr 2020	Georgia	• The user will be given a unique ID that allows the app to anonymously determine social interactions with other app users. Encounters that have a certain intensity in terms of time and proximity are stored locally by both apps in an encrypted form. If someone tests positive for COVID-19, people who may have had contact with the infected person within the last few days will receive a warning with instructions to contact the local governmental authority.	Ministry of Idps, Labour, Health and Social Affairs	https://apps.apple.com/sa/app/stop-covid/id1507435357
53.	StopKorona!	12 Apr 2020	North Macedonia	• Detect the distance between other people's mobile devices / applications<br>• The main purpose of the application is to provide a prompt response to the health authorities for persons who have been in close contact with the infected person in the past 14 days.	Ministry of Health (North Macedonia), Nextsense	https://apps.apple.com/sa/app/stopkorona/id1506641869
54.	Kencor Covid-19	13 Apr 2020	US	• Check symptoms towards COVID-19. It can be integrated with Apple Health Kit, and if the user decides to allow the app with the access, basic vital/fitness information will be gathered and provided to the healthcare provider.<br>• Allows the hospital health officials to see the trend in the community to take meaningful measures to handle the spread of the virus	Kencor Health, Inc.	https://apps.apple.com/sa/app/kencor-covid-19/id1507108440
55.	NHS24 Covid-19	13 Apr 2020	UK	• Symptom checker that will assess your symptoms and provide some guidance as what to do next.<br>• Instant access to quality assured COVID-19 information from NHS 24·	NHS 24	https://apps.apple.com/sa/app/nhs24-covid-19/id1506916940
56.	PeduliLindungi (Care Protect)	13 Apr 2020	Indonesia	• This application relies on caring (caring) and community participation to share location data with each other while traveling so that tracing of the contact history with sufferers of COVID-19 can be done. Caring Protect uses your Bluetooth to record the information needed. Data exchange will occur when there are other gadgets within the Bluetooth radius that are also registered with CareCare. Care Protect will then identify people who have been in close proximity to people who tested positive for COVID-19 or PDP (Patients Under Supervision) and ODP (People in Supervision). You will also be contacted by a health worker if you have been within a certain distance with a positive COVID-19, PDP, and ODP sufferer.	Indonesian Ministry of Communication and Information Technology	https://apps.apple.com/sa/app/pedulilindungi/id1504600374
57.	Hayat Eve Sığar	14 Apr 2020	Turkey	• It will inform and guide our citizens about the New Coronavirus (Covid-19).<br>• It shows basic need points such as hospitals, pharmacies, market chains, subways and stops on the map, you can see the density of isolation at home, infected people and risky areas	T.R. Ministry of Health	https://apps.apple.com/sa/app/hayat-eve-s%C4%B1%C4%9Far/id1505756398
58.	Apollo Covid-19	16 Apr 2020	US	• Apollo helps people determine if they are likely to qualify for COVID-19 testing based on CDC guidelines and helps them find a nearby testing facility from a nationwide directory.	Gauss Surgical, Inc.	https://apps.apple.com/sa/app/apollo-covid-

						19/id1503695588
59.	Covid Coach	17 Apr 2020	US	• Connect you to important resources for coping and adapting during the COVID-19 pandemic.<br>• Customized tools are available to help you cope with stress, stay well, stay safe, stay healthy, stay connected, and navigate parenting, care-giving, and working from home while social distancing, quarantined, or sheltered in place.<br>• Track your mood, visualize your progress, and find resources to seek additional help and support.	Department of Veterans Affairs (VA) in US	https://apps.apple.com/sa/app/covid-coach/id1504705038
60.	Bluezone - Electronic Mask	18 Apr 2020	Vietnam	• Bluezone alert if user had close contact with people who have COVID-19	Ministry of Information and Communications & Ministry of Health	https://apps.apple.com/vn/app/bluezone/id1508062685?ls=1
61.	Covid Radar.MX	19 Apr 2020	Mexico	• Help detect and alert everyone they have been with in contact in the last 14 days, both directly and indirectly (random people) notifying them of this situation, guaranteeing in any case the anonymity. (Translated)	Government of the State of Nuevo León	https://apps.apple.com/sa/app/covidradar-mx/id1508215043
62.	Jamcovid19	23 Apr 2020	Jamaica	• Self-reporting and monitoring portal. Any person who entered Jamaica on or after 18th of March is mandated by the Ministry of Health to report through this APP.<br>• Latest data and statistics<br>• Direct links to Emergency Services along with other critical functionalities.	Office of The Prime Minister, Jamaica	https://apps.apple.com/sa/app/jamcovid19/id1509638185
63.	MyAus Covid-19	23 Apr 2020	Australia	• Information about COVID-19 and how it impacts you in Australia.	Migration Council Australia	https://apps.apple.com/sa/app/myaus-covid-19/id1508274448
64.	Plan Jalisco Covid-19	25 Apr 2020	Mexico	• Verifies with the authorities if users have had contact with people suffering from the coronavirus and/or have been exposed to conditions and/or places that could represent a "contact close" with the virus, and allows the authorities to establish the proper contact and follow-up of the cases of those people suspected and/or infected by COVID-19, and achieve a more agile and effective response in each particular case.<br>• Provides information on the symptoms and/or prevention of the COVID-19 virus in order to help prevent its spread, and to be able to detect if they are being affected by the disease and obtain recommendations.	Secretary of Finance, The National health organisation/ Government of The State of Jalisco	https://apps.apple.com/us/app/plan-jalisco-covid-19/id1504356187
65.	Talli Symptom Tracker	25 Apr 2020	US	• Keep track of symptoms relevant to COVID-19 and share with your doctor to follow. You can manage the list of medications available for each person you are tracking for.	Babylogger LLC	https://apps.apple.com/sa/app/talli-symptom-tracker/id1505549920
66.	EHTERAZ	25 Apr 2020	Qatar	• The latest updates of COVID-19 Coronavirus in Qatar to spread the health awareness tips and techniques as well as the protection methods that are necessary to halt the outbreak of Coronavirus.	Ministry of Interior	https://apps.apple.com/sa/app/ehteraz/id1507150431
67.	COVIDSafe	26 Apr 2020	Australia	• Take a note of contact you've had with other users by securely logging the other user's reference code<br>• If you or someone you've been in contact with is diagnosed with COVID-19, the close contact information securely stored in your phone can be uploaded and used—with your consent—by state and territory health officials to quickly inform people who've been exposed to the virus.	Australian Department of Health	https://apps.apple.com/sa/app/covidsafe/id1509242894
68.	Covid AI	26 Apr 2020	US	• It is an artificial intelligence app designed to ease screening for radiologists and clinicians. COVID-AI app data satisfies HIPAA and DICOM compliance standards.	AI Biokinetic Technologies	https://apps.apple.com/sa/app/covid-ai/id1505887668
69.	Covid-19 Virginia Resource	27 Apr 2020	US	• latest news and updates on COVID-19.<br>• Search for local resources, Virginia's online system to apply for assistance.<br>• Vital information and guidance from other agencies throughout the Commonwealth of Virginia.	Virginia Department of Social Services	https://apps.apple.com/sa/app/covid-19-virginia-resources/id1507112717

70.	Odisha Covid Dashboard	28 Apr 2020	Odisha, India	• Informative app for citizens regarding Corona status.	Odisha Computer Application Centre	https://apps.apple.com/sa/app/odisha-covid-dashboard/id1503472515
71.	NIOSH PPE Tracker	29 Apr 2020	US	• To assist facilities in planning and optimizing the use of PPE resources for the response to coronavirus disease 2019 (COVID-19). It allows calculating the number of boxes in hospitals' stocks such as gowns, surgical masks, respirators, face shields to find the average consumption rate.	Centers for Disease Control and Prevention (US)	https://apps.apple.com/sa/app/niosh-ppe-tracker/id1506310638
72.	ProteGO Safe	30 Apr 2020	Poland	• Notifications about possible contact with coronavirus.<br>• Perform a risk assessment test. The risk assessment test is a simple survey created by doctors based on the guidelines of the World Health Organization and the Chief Sanitary Inspectorate.<br>• Receive a behavioral recommendation.<br>• You will learn what to do next and where to look for help if needed.<br>• keep a health journal, which is a regular record of information about how you feel and your condition.<br>• The health diary is a tool helpful for doctors. Facilitates providing medical personnel with information about the development of symptoms, concomitant diseases or medications taken. you will get a chance for a faster diagnosis and choosing the right treatment regimen.	Ministry of Digital Affairs of Poland	https://apps.apple.com/sa/app/protego-safe/id1508481566
73.	eRouška	3 May 2020	Czech Republic	• The app allows you to send an anonymous list of eRouška users with whom you have been in risky contact with one click including those you don't know personally, if you just stood in line in the store, or just took a bus with them. No one will know that you may have infected them.	Czech Ministry of Health and Hygiene	https://apps.apple.com/sa/app/erou%C5%A1ka/id1509210215
74.	PathCheck SafePlaces	3 May 2020	Global	• It aids in COVID-19 exposure notification and contract tracing.	PathCheck Foundation	https://apps.apple.com/us/app/covid-safe-paths/id1508266966
75.	Cova Punjab	8 May 2020	Pakistan	• Provides preventive care information.<br>• Helpline.<br>• Real time dashboard for Punjab, India and global stats<br>• Push notifications provide updates from government, advisories and instructions from time to time.	Government of Punjab	https://apps.apple.com/in/app/cova-punjab/id1501977319
76.	NOVID	11 May 2020	US	• Early Warning information about disease spread relative to the network of people you interact with.<br>• It also gives you real-time feedback on social distancing, The NOVID interface shows you how far away it estimates other devices to be, so that you can know it is working accurately<br>• It also gives you exposure alerts if you have spent a long-time near people who later self-report positive tests.	Expii, CMU	https://apps.apple.com/sa/app/novid/id1508029182
77.	CovTracer	15 May 2020	Republic of Cyprus	• It checks the user's location trails, and identify the places a carrier has visited and in turn, locate other citizens who have been in close proximity to the diagnosed carrier, thus acting to reduce the spread of COVID-19.	Deputy Ministry for Research, Innovation & Digital Policy	https://apps.apple.com/sa/app/covtracer/id1510330601
78.	NZ COVID Tracer	19 May 2020	New Zealand	• Enable faster contact tracing. Create a digital diary of the places you visit by scanning the official QR codes at the entrances to business premises, other organizations and public buildings. Share your digital diary with contact tracers if you're found to have COVID-19.<br>• Sign up for contact alerts in case you've checked into a location at the same time as someone with COVID-19.	Ministry of Health	https://apps.apple.com/sa/app/nz-covid-tracer/id1511667597
79.	CDC	21 May 2019	USA	• Getting the most up to date health information.<br>• Top of health news in the Newsroom section, and view CDC Images of the Week. Variety of content such as stories, videos, podcasts, and features to give you the most current health information from CDC.<br>• If you are a journal reader, view the latest Morbidity & Mortality Weekly Report, Emerging and Infectious Disease journal, or the latest on Preventing Chronic Diseases.	Centers for Disease Control and Prevention (US)	https://apps.apple.com/us/app/cdc/id487847188
80.	Wiqaytna (Our prevention)	26 May 2020	Morocco	• It allows mobile phones for its users to monitor other phones aligned using the same application, and to share encrypted and anonymous identification codes through these devices. In the event that it is confirmed that a user of this application has been infected with the emerging coronavirus, which causes Covid-19 disease. The health authorities ask him to send the list of	Ministry of Interior	https://apps.apple.com/sa/app/%D9%88%D9%82%D8%A7%D9%8A%D8%AA%D9%86%D

				• identification codes stored locally on the level of his mobile phone to a central information server, using the application "data retransmission".<br>• Enable the identification of people who were present near the affected person, via an SMS.		8%A7/id1512666410
81.	Apturi Covid	28 May 2020	Latvia	• The app will notify you if you have had contact with a Covid-19 sufferer - without revealing the identity of either the sender or the recipient.<br>• It will also arm you with the knowledge of what to do if you receive such a message.	Consortium of volunteers, Ministry of Health of the Republic of Latvia	https://apps.apple.com/sa/app/apturi-covid-latvia-spkc/id1513573144
82.	VírusRadar	29 May 2020	Hungary	• Detecting the distance of mobile devices / applications using Bluetooth<br>• The app is designed to allow epidemiologists to respond quickly if someone has been in contact with an infected person in the past 14 days.<br>• If a user becomes infected with the virus, the Hungarian State will ask you to share your information with epidemiologists, who will identify the telephone numbers of other users who have been in contact with them and notify them that they have been in contact with a COVID-19 infected person. without disclosing your details.	Ministry of Innovation and Technology	https://apps.apple.com/sa/app/v%C3%ADrusradar/id1511740371
83.	Spectrum	29 May 2019	US	• Spectrum is a customizable clinical decisions tool for infectious diseases that includes COVID-19 guidelines, Infection Prevention & Control protocols, etc.	Spectrum Mobile Health Inc.	https://apps.apple.com/sa/app/spectrum-clinical-decisions/id921339941
84.	Immuni	1 Jun 2020	Italy	• It will notify users at risk of carrying the virus as early as possible—even when they are asymptomatic.	Presidenza del Consiglio dei Ministri	https://apps.apple.com/sa/app/immuni/id1513940977
85.	StopCovid	2 Jun 2020	France	• If you take a test for COVID-19 that turns out to be positive, the laboratory will give you a code to scan or enter manually to send an anonymous alert to users who have been close to you.	Government of France	https://apps.apple.com/sa/app/stopcovid-france/id1511279125
86.	SwissCovid	2 Jun 2020	Switzerland	• Contact tracing carried out by the cantons. If a SwissCovid app user tests positive for the coronavirus, the cantonal authorities issues them with a code (the Covidcode). The code allows them to activate the notification function in their app. This warns other app users that have had an encounter with the infected person in the period starting two days before that person first experienced symptoms of the disease). When the code is entered, the app notifies these other app users automatically and anonymously.	Ubique, EPFL, ETH Zurich	https://apps.apple.com/sa/app/swisscovid/id1509275381
87.	E7mi	2 Jun 2020	Tunisia	• Once the application is downloaded and installed, it automatically begins to detect and store the interactions that have occurred, that is to say, whenever you are within a radius of a few meters from another person with the application, without revealing the user's identity in the context of respecting personal data.	National Observatory for New and Emerging Diseases	https://apps.apple.com/sa/app/e7mi-%D8%A5%D8%AD%D9%85%D9%8A/id1513856060
88.	Self-Shield (Formerly COVID Shield)	9 Jun 2020	Sri Lanka	• It can support you keep track of your health.<br>• It uses advance AI based analytics to assess the symptoms that you disclose, existing medical conditions, your state of breathing and other data to determine your health and keep track of your progress.<br>• enable you to enroll into a Self-Shield Program, when such program is available in your country, and obtain medical care based on your need. This will avoid unnecessary delays and will help healthcare teams to respond quickly and efficiently, and reward users for good behavior as they engage in self-health checking on a regular basis.	Commonwealth Centre for Digital Health	https://apps.apple.com/sa/app/self-shield/id1514207089
89.	Tabaud Saudi	12 Jun 2020	KSA	• Notifying people if they had contact with others confirmed to be infected with coronavirus;<br>• Providing them help by sending their health forms to the Ministry of Health to provide them necessary medical support according to the status and progress of the case.<br>• Enabling those confirmed to be infected with coronavirus to voluntarily share their tests' results with people they had contact with during the past 14 days.	National Information Center NIC, & Data and Artificial Intelligence Authority SDAIA, in close	https://apps.apple.com/sa/app/tabaud-covid-19-ksa/id1514704802

					cooperation with the Ministry of Health MoH	
90.	CareFIJI	14 Jun 2020	Republic of Fiji	• It assists the Ministry of Health and Medical Services streamline and speed up its manual contact tracing efforts. careFIJI uses Bluetooth signals to determine if you are near other careFIJI users. This close contact data is anonymised, encrypted and stored on your mobile phones. Only a mobile number is required to activate the careFIJI app and Ministry of Health and Medical Services officials will use the same number to call you to conduct contact tracing.	Government of the Republic of Fiji	https://apps.apple.com/sa/app/carefiji/id1513752467
91.	Pre Work Screen	16 Jun 2020	US	• Employee COVID-19 Self-screen.	InfoBeyond Technology LLC	https://apps.apple.com/sa/app/preworkscreen/id1517912375
92.	Smitte|stop	17 Jun 2020	Denmark	• It allows you to be notified when you have been close to another user of the app who has received COVID-19.<br>• You can notify other users of the app if you are tested positive. They can't see who you are.	Ministry of Health (Denmark)	https://apps.apple.com/sa/app/smitte-stop/id1516581736
93.	ProjectCovid	1 Jul 2020	USA	• Live Global Outbreak Tracker: Find up-to-date statistics about the spread of COVID-19 in your state, country, and around the world.<br>• Latest News: A hub of breaking news stories, vetted information, and general guidelines to keep you updated on COVID-related events around the world.<br>• Global Resources: Our global resources section features an information toolkit, symptom check platform, preventative practices and mental health resources.<br>• Testing Center Information: This section consists of contact info (contact, and email) by state, and it provides a step-by-step guide on finding and availing testing services around the country.	LFR International	https://apps.apple.com/sa/app/projectcovid-verified-info/id1505613126
94.	Radar COVID	7 Jul 2020	Spain	• Alerts you anonymously of the possible contact that you have had in the last 14 days with a person who has been infected.<br>• allows: Anonymously communicate your positive diagnosis. Communicate the exhibition anonymously to the people with whom you have been in contact.	Ministry of Economic Affairs and Digital Transformation, Indra Sistemas	https://apps.apple.com/sa/app/radar-covid/id1520443509
95.	COVID Alert	30 Jul 2020	Canada	• COVID Alert uses Bluetooth to exchange random codes with nearby phones. It does not use or access any location data. COVID Alert works by determining how far away other phones are by the strength of their Bluetooth signal.	Health Canada	https://apps.apple.com/sa/app/covid-alert/id1520284227
96.	COVIDWISE	5 Aug 2020	USA	• COVIDWISE is the official COVID-19 exposure notification app for the Commonwealth of Virginia's Department of Health (VDH). The app was developed in partnership with SpringML using a Bluetooth Low Energy (BLE) API framework created through a unique collaboration between Apple and Google.	Virginia Department of Health	https://apps.apple.com/sa/app/covidwise/id1518059690
97.	CoronaMelder	11 Aug 2020	Netherlands	• The app will warn you after you have been close to someone with the corona virus. CoronaMelder sees via Bluetooth when you are close to other people with the app. The app does not use any personal or location data. And do not know who you are and where you were.	The Ministry of Health, Welfare and Sport	https://apps.apple.com/nl/app/id1517652429
98.	GuideSafe	17 Aug 2020	USA	• Exposure Notification app to anonymously share a positive COVID-19 test result — and be anonymously notified of your own possible exposure to someone who later reports a positive COVID-19 test result — all without sharing anyone's identity.	Alabama Department of Public Health	https://apps.apple.com/sa/app/guidesafe/id1519514691
99.	Covid Watch Arizona	18 Aug 2020	USA	• Let your smartphone notify you to potential exposure to COVID-19–using fully anonymous Bluetooth signals–and help stop the spread of coronavirus in Arizona.	Arizona Department of Health Services	https://apps.apple.com/sa/app/covid-watch-arizona/id1521655110
100	Protect Scotland	10 Sep 2020	UK	• When you use the app, you will be alerted if you have been in close contact with another app user who has tested positive for coronavirus. If you test positive and you enter the unique code sent to you into the app, it will anonymously warn other app users whom you have been in close contact with.	NHS Education for Scotland	https://apps.apple.com/gb/app/id1526637715

101	VoxCovid	Not available	France	• Allows an easy update of the datasets initially collected through the app VoxInfra.<br>• Map this is useful to conduct community-based monitoring in countries post conflict, in particular Afghanistan.	Delesgues Solutions	https://apps.apple.com/sa/app/voxcovid/id1506196683
102	Estamos on – covid19	Not available	Portuguese	• Advice for working and using public services from home, FAQs is also available, collected on social networks and answered, with reliable information, and the recommendations of the Health authorities.<br>• Statistics of the evolution of the pandemic in Portugal. (Translated)	Agencia Para A Modernizacao Administrrativa	https://apps.apple.com/sa/app/estamos-on-covid19/id1502916368
103	Covid Asist	Not available	Romania	• Users can self-monitor their general health in the context of the SARS - VOC 2 coronavirus pandemic, in order to regularly monitor the evolution of symptoms specific to COVID- 19 disease.<br>• Offers digitized support to the medical system, by remote monitoring of an extended number of patients.<br>• The application generates information messages on the general state of health or alerts of recommendation contracting the specialized medical staff. (Translated)	National Institute of Infectious Diseases	https://apps.apple.com/sa/app/covid-asist/id1509275772
104	InfoCovid	Not available	Italy	• The user asks InfoCOVID a question: 'Can I move to go to work?'. InfoCOVID provides the FAQ most similar to the question such as presenting the FAQ on containment measures and the relative authorization form for travel. InfoCOVID sorts the FAQ by relevance, that is 'similarity' to the question posed, and presents them to the citizen. InfoCOVID guarantees always certified answers: the FAQs are entered and validated by domain experts. (Translated)	Question Cube	https://apps.apple.com/sa/app/infocovid/id1504909827
105	WHO Info	Not available	Global	• The latest news, events, features<br>• Breaking updates on outbreaks.	World Health Organization WHO	https://play.google.com/store/apps/details?id=org.who.infoapp
106	Corona Map	Not available	KSA	• Interactive map application that allows users to track all coronavirus cases in the word<br>• Statistics and charts<br>• The ability to talk to BashairBot to answer questions about CoronaVirus.	National Health Information Center	https://play.google.com/store/apps/details?id=com.CoronaMapSA
107	Tawakkalna KSA	Not available	KSA	• Instant and live information about the number of coronavirus infections in the Kingdom<br>• It allows citizens and residents to request movement permits in cases of necessity during curfew; follow-up on their permit request status during curfew; and notify them when they are close to infectious or isolated areas.<br>• Report COVID-19 suspected cases to help individuals receive the health care they, or others, need.	National Information Center	https://play.google.com/store/apps/details?id=sa.gov.nic.tawakkalna
108	تطمن Tatamman	Not available	KSA	• The results of the COVID-19 examinations<br>• Contact 937 and ask for help<br>• Daily health questionnaire<br>• Update contact information<br>• Educational Content Library<br>• Countdown indicator for self-isolation<br>• Alerts with notifications, text messages and automatic calls	Ministry of Health	http://play.google.com/store/apps/details?id=com.tetaman.home
109	AMAN (Safety)	Not available	Jordan	• If someone is diagnosed with coronavirus by the Ministry of Health, the people who have been recently in contact with her/him will be notified through the app advising them to isolate and call 111, MOH’s COVID-19 Hotline, for instructions. The app uses anonymous GPS data to detect exposure to COVID-19 patients.	Jordan's Ministry of Health	https://apps.apple.com/sa/app/aman-aman-jo-jordan-covid-19/id1511595289
110	MyTrace	Not available	Malaysia	• Participating devices exchange contact information whenever an app detects another nearby device with MyTrace installed. The app enables identification of people who have been in close proximity to an infected person.	Government of Malaysia	https://apps.apple.com/sa/app/mytrace/id1508140938
111	Sehet Misr (Egypt’s health)	Not available	Egypt	• The app collects the GPS location of the phone and stores locally on the device. If the phone owner is diagnosed with the Covid-19 disease he is asked to share the location data for contact tracing in order to identify individuals that might need to go into quarantine.	Health Ministry	https://play.google.com/store/apps/details?id=eg.com.eserve.sehatmisr&hl=en_US&gl=US

112	Self-Diagnosis app	Not available	South Korea	• Record their daily health status on the app for 14 days after arrival in Korea.	Ministry of Health and Welfare	https://play.google.com/store/apps/details?id=com.mohw.corona&hl=en
113	Self-Quarantine app	Not available	South Korea	• Diagnosis function of self-container (notification of diagnosis time, submission of diagnosis result)<br>• Self-container safety rules information providing function<br>• Emergency contact network function of self-contained person (dedicated official phone number)	Ministry of the Interior and Safety	https://play.google.com/store/apps/details?id=kr.go.safekorea.sqsm&hl=en
114	Private Kit: Safe Paths	Not available	US	• It allows you to log your location once every five minutes and identify areas of overlap with public time-stamped GPS location data. You can also import prior location history from other apps to quickly build your location history and start comparing for GPS overlap.	MIT	https://play.google.com/store/apps/details?id=edu.mit.privatekit&hl=en
115	Coalition App	Not available	Global	• App alerts you if you have crossed paths or spent time with another user showing symptoms, or those who have tested positive for the virus. Instead of using your location data, Coalition uses secured Bluetooth to protect your privacy. Your information stays anonymous and private, and isn't shared with anyone without your permission.<br>• Only when you confirm that you are sick, does Coalition share an anonymous ID with others who you have come into contact with.	Coalition Network	https://play.google.com/store/apps/details?id=world.coalition.app&hl=en

Note: The app's release date is not available for apps in the Google Play and for apps that have no updated versions in the Apple Store as per 11 Sep 2020.
