# Supplementary material for: Health Apps for Combating COVID-19: Descriptive Review and Taxonomy
Source: JMIR Mhealth Uhealth. 2021 Mar 2;9(3):e24322. doi: 10.2196/24322 (PMC7927949; doi:10.2196/24322)
Supplement: Multimedia Appendix 2 [file mhealth_v9i3e24322_app2.pdf]

Appendix 2

The table below presents the occurrence of excerpts in each app included in this review.

	Technical Features (Codes)																														
App Name (N=109)	Alert contacts	Basic health information and advice or FAQ	Chatbot	Checklist of surfaces that have to be disinfected	Contact tracing	Distance detection	Gadget of self-assessment	Health or travel declaration	Helpline	Information about health services and care lines	Latest news	List of products	Live statistics and rolling updates (Push notifications)	Live statistics and rolling updates (RSS feeds)	Location monitoring	Making medical appointments	Map	Medical check-up tracking	Medical report generator	Medication tracking and reminders	Mood tracking and mental status	Movement permits	Recruitment of volunteers	Remote monitoring	Results of Covid-19 laboratory test	Sharing data or story with others	Symptoms tracker	Taking photos of surfaces	Virtual medical consultation	Wearable devices for symptoms tracking	Totals
Covid-19 Czechia	0	1	0	0	0	0	0	0	0	0	1	0	0	1	0	0	1	0	0	0	0	0	0	0	0	0	0	0	0	0	4
TraceCovid	0	0	0	0	1	0	0	0	0	0	0	0	0	0	0	0	0	0	0	0	0	0	0	0	0	0	0	0	0	0	1
HealthLynked COVID-19 Tracker	1	0	1	0	0	0	1	0	0	0	1	0	0	0	0	0	1	0	0	0	0	0	0	0	0	0	1	0	0	0	6
Covid-19 Vietnam	0	1	1	0	0	0	0	1	0	1	1	0	0	0	0	0	1	0	0	0	0	0	0	0	0	0	0	0	1	0	7
Bluezone	1	0	0	0	0	0	0	0	0	0	0	0	0	0	0	0	0	0	0	0	0	0	0	0	0	0	0	0	0	0	1
COVID-19 Medisch Dossier	0	1	0	0	0	0	0	0	0	0	0	0	1	1	0	0	0	0	0	0	0	0	0	0	0	0	0	0	0	0	3
Corona FACTS	0	1	0	0	0	0	0	0	0	0	1	0	0	0	0	0	1	0	0	0	0	0	0	0	0	1	0	0	0	0	4
Plan Jalisco Covid-19	0	1	0	0	1	0	0	0	0	0	0	0	0	0	0	0	0	0	0	0	0	0	0	0	0	0	0	0	0	0	2
PatientMpower for COVID-19	0	0	0	0	0	0	0	0	0	0	0	0	0	0	1	0	0	0	0	0	0	0	0	1	0	0	0	0	0	1	3
CDC	0	1	0	0	0	0	0	0	0	0	1	0	1	0	0	0	0	0	0	0	0	0	0	0	0	0	0	0	0	0	3
Coronavirus-SUS	0	1	0	0	0	0	0	0	0	0	1	0	0	0	0	0	1	0	0	0	0	0	0	0	0	0	0	0	0	0	3
Cova Punjab	0	1	0	0	0	0	0	0	1	0	0	0	1	1	0	0	0	0	0	0	0	0	0	0	0	0	0	0	0	0	4
Disinfection Checklist	0	0	0	1	0	0	0	0	0	0	0	1	0	0	0	0	0	0	0	0	0	0	0	0	0	0	0	1	0	0	3
NCOVI	0	1	0	0	0	0	0	1	0	0	0	0	0	1	0	0	0	0	0	0	0	0	0	0	0	0	0	0	0	0	3
Patientsphere for Covid19	0	0	0	0	0	0	0	0	0	0	0	0	0	0	0	0	0	0	0	1	0	0	0	0	0	1	1	0	0	0	3
Coronavirus Australia	0	1	0	0	0	0	1	0	0	0	1	0	1	0	0	0	0	0	0	0	0	0	0	0	0	0	0	0	0	0	4
Coronavirus UY	0	0	0	0	0	0	1	0	0	0	0	0	0	0	0	0	0	0	0	0	0	0	0	0	0	0	0	0	0	0	1
Covid-19 UAE	0	0	0	0	0	0	0	0	1	0	1	0	0	0	0	0	0	0	0	0	0	0	0	0	0	0	0	0	0	0	2
Stop Covid19	0	0	0	0	0	0	1	0	0	0	0	0	0	0	0	0	1	0	0	0	0	0	0	0	0	0	0	0	0	0	2
Canada Covid-19	0	1	0	0	0	0	0	0	0	0	1	0	0	0	0	0	0	0	0	0	0	0	0	0	0	0	0	0	0	0	2
AarogyaSetu	0	1	0	0	0	0	0	0	0	0	0	0	0	0	0	0	0	0	0	0	0	0	0	0	0	0	0	0	0	0	1
Covid-19 Armenia	0	0	0	0	0	0	1	0	0	0	1	0	0	0	0	0	0	0	0	0	0	0	0	0	0	0	0	0	0	0	2
Family-Covid19	0	0	0	0	0	0	0	1	0	0	0	0	0	0	0	0	0	0	0	0	0	0	0	0	0	0	0	0	0	0	1
Trecovid19	0	1	0	0	0	0	0	0	0	0	0	0	0	0	0	0	0	0	0	0	0	0	0	0	0	0	0	0	0	0	1
Covive	0	1	0	0	0	0	1	0	0	0	0	0	0	0	0	0	0	0	0	0	0	0	0	0	0	0	1	0	0	0	3

Bolivia Segura	0	1	0	0	0	0	1	0	0	0	1	0	0	1	1	0	0	0	0	0	0	0	0	0	0	0	1	0	0	0	6
Covidom Patient	0	0	0	0	0	0	0	0	0	0	0	0	0	0	0	0	0	0	0	0	0	0	0	1	0	0	0	0	0	0	1
Nua health Video Consultations	0	0	0	0	0	0	0	0	0	0	0	0	0	0	0	0	0	0	0	0	0	0	0	0	0	0	0	0	1	0	1
Laziodr Covid	0	0	0	0	0	0	1	0	1	0	0	0	0	0	0	0	0	0	0	0	0	0	0	0	0	0	0	0	1	0	3
Castor covid-19	0	0	0	0	0	0	0	0	0	0	0	0	0	0	1	0	0	0	0	0	0	0	1	0	0	0	1	0	0	0	3
Covid19-dxb Smart App	0	1	0	0	0	0	0	0	0	0	0	0	1	0	0	0	0	0	0	0	0	0	0	0	0	0	0	0	1	0	3
Stop Covid	0	0	0	0	1	0	0	0	0	0	0	0	0	0	0	0	0	0	0	0	0	0	0	0	0	0	0	0	0	0	1
Apollo Covid-19	0	0	0	0	0	0	1	0	0	0	0	0	0	0	0	0	0	0	0	0	0	0	0	0	0	0	0	0	0	0	1
Covid Radar	0	0	0	0	0	0	0	0	0	0	0	0	0	0	0	0	0	0	0	0	0	0	1	0	0	0	0	0	0	0	1
SOS CORONAVIRUS	0	1	0	0	0	0	0	0	0	0	0	0	0	0	0	0	0	0	0	0	0	0	0	0	0	0	0	0	0	0	1
CoronApp Colombia	0	0	0	0	0	0	1	1	0	1	0	0	0	1	1	0	0	0	0	0	0	0	0	0	0	0	0	0	0	0	5
Premedicus	0	1	0	0	0	0	1	0	0	1	0	0	0	0	0	0	0	0	1	0	0	0	0	0	0	0	0	0	1	0	5
Covid-19 Virginia Resource	0	1	0	0	0	0	0	0	0	1	1	0	0	0	0	0	0	0	0	0	0	0	0	0	0	0	0	0	0	0	3
Covid Coach	0	0	0	0	0	0	0	0	0	1	0	0	0	0	0	0	0	0	0	0	1	0	0	0	0	0	0	0	0	0	2
BC Covid-19	0	1	0	0	0	0	0	0	0	0	1	0	0	0	0	0	0	0	0	0	0	0	0	0	0	0	0	0	0	0	2
GVA CoronVirus	0	1	0	0	0	0	0	0	0	0	0	0	0	0	0	1	0	0	0	0	0	0	0	0	0	0	0	0	0	0	2
Corona Care	0	0	1	0	0	0	0	0	0	0	0	0	0	0	0	0	0	0	0	0	0	0	0	0	0	0	1	0	1	0	3
VoxCovid	0	0	0	0	0	0	0	0	0	0	0	0	0	0	0	0	1	0	0	0	0	0	0	0	0	1	0	0	0	0	2
Jamcovid19	0	0	0	0	0	0	0	1	0	1	1	0	0	0	0	0	0	0	0	0	0	0	0	0	0	0	0	0	0	0	3
Estamos on Covid19	0	1	0	0	0	0	0	0	0	0	0	0	0	1	0	0	0	0	0	0	0	0	0	0	0	0	0	0	0	0	2
Odisha Covid Dashboard	0	1	0	0	0	0	0	0	0	0	0	0	0	0	0	0	0	0	0	0	0	0	0	0	0	0	0	0	0	0	1
Covid Watcher	0	0	0	0	0	0	0	0	0	0	0	0	0	0	1	0	1	0	0	0	0	0	0	0	0	1	0	0	0	0	3
Kencor Covid-19	0	0	0	0	0	0	0	0	0	0	0	0	0	0	0	0	0	0	0	0	0	0	0	1	0	0	1	0	0	0	2
MyAus Covid-19	0	1	0	0	0	0	0	0	0	0	0	0	0	0	0	0	0	0	0	0	0	0	0	0	0	0	0	0	0	0	1
NHS24 Covid-19	0	1	0	0	0	0	0	0	0	0	0	0	0	0	0	0	0	0	0	0	0	0	0	0	0	0	0	0	0	0	1
Talli Symptom Tracker	0	0	0	0	0	0	0	0	0	0	0	0	0	0	0	0	0	0	0	0	0	0	0	0	0	0	1	0	0	0	1
Covid Radar.MX	1	0	0	0	0	0	0	0	0	0	0	0	0	0	0	0	0	0	0	0	0	0	0	0	0	0	0	0	0	0	1
Covid Asist	0	1	0	0	0	0	0	0	0	0	0	0	0	0	0	0	0	0	0	0	0	0	0	1	0	0	1	0	0	0	3
InfoCovid	0	1	0	0	0	0	0	0	0	0	0	0	0	0	0	0	0	0	0	0	0	0	0	0	0	0	0	0	0	0	1
COVID-19 TAM	0	0	0	0	0	0	0	0	0	0	0	0	0	1	1	0	0	0	0	0	0	0	0	0	0	0	0	0	0	0	2
BMC Combat COVID-19	0	0	0	0	0	0	0	0	0	0	0	0	0	0	0	0	0	0	0	0	0	0	0	0	0	0	1	0	0	0	1
Rakning C-19	0	0	0	0	1	0	0	0	0	0	0	0	0	0	0	0	0	0	0	0	0	0	0	0	0	0	0	0	0	0	1
COVID Puebla	0	0	0	0	0	0	1	0	0	0	0	0	0	0	0	0	0	0	0	0	0	0	0	0	0	0	0	0	0	0	1
Asistencia COVID-19	0	0	0	0	1	0	1	0	0	0	0	0	0	0	0	0	0	0	0	0	0	0	0	0	0	0	0	0	0	0	2
Tabaud KSA	1	0	0	0	1	0	0	0	0	1	0	0	0	0	0	0	0	0	0	0	0	0	0	0	0	0	0	0	0	0	3
WHO Info	0	0	0	0	0	0	0	0	0	0	1	0	0	1	0	0	0	0	0	0	0	0	0	0	0	0	0	0	0	0	2
Corona Map	0	0	1	0	0	0	0	0	0	0	0	0	0	1	0	0	1	0	0	0	0	0	0	0	0	0	0	0	0	0	3
Tawakkalna Saudi	0	0	0	0	0	0	0	1	0	0	0	0	0	1	0	0	0	0	0	0	0	1	0	0	0	0	0	0	0	0	3
Tatamman تطمن	1	1	0	0	1	0	2	0	1	0	0	0	0	0	0	0	0	0	0	0	0	0	0	0	1	0	0	0	0	0	7
COVIDSafe	1	0	0	0	1	0	0	0	0	0	0	0	0	0	0	0	0	0	0	0	0	0	0	0	0	0	0	0	0	0	2
Stopp Corona	1	1	0	0	1	0	0	1	0	0	0	0	0	0	0	0	0	0	0	0	0	0	0	0	0	0	1	0	0	0	5
eRouška	1	0	0	0	1	0	0	0	0	0	0	0	0	0	0	0	0	0	0	0	0	0	0	0	0	0	0	0	0	0	2
smitte|stop	1	0	0	0	1	0	0	0	0	0	0	0	0	0	0	0	0	0	0	0	0	0	0	0	0	0	0	0	0	0	2

StopCovid	1	0	0	0	0	0	0	0	0	0	0	0	0	0	0	0	0	0	0	0	0	0	0	0	0	0	0	0	0	0	1
VírusRadar	1	0	0	0	1	1	0	0	0	0	0	0	0	0	0	0	0	0	0	0	0	0	0	0	0	0	0	0	0	0	3
Aarogya Setu	0	1	0	0	0	0	0	0	0	0	0	0	0	0	0	0	0	0	0	0	0	0	0	0	0	0	0	0	0	0	1
GoK Direct	0	0	0	0	0	0	0	0	0	1	0	0	0	1	0	0	0	0	0	0	0	0	0	0	0	0	0	0	0	0	2
PeduliLindungi (Care Protect)	0	0	0	0	1	0	0	0	0	0	0	0	0	0	0	0	0	0	0	0	0	0	0	0	0	0	0	0	0	0	1
Immuni	1	0	0	0	0	0	0	0	0	0	0	0	0	0	0	0	0	0	0	0	0	0	0	0	0	0	0	0	0	0	1
AMAN (أمان Safety)	0	0	0	0	1	0	0	0	0	0	0	0	0	0	0	0	0	0	0	0	0	0	0	0	0	0	0	0	0	0	1
Apturi Covid	1	1	0	0	0	0	0	0	0	0	0	0	0	0	0	0	0	0	0	0	0	0	0	0	0	0	0	0	0	0	2
MySejahtera	0	0	0	0	0	0	1	0	0	0	0	0	0	0	0	0	0	0	0	0	0	0	0	1	0	0	1	0	0	0	3
MyTrace	1	0	0	0	1	0	0	0	0	0	0	0	0	0	0	0	0	0	0	0	0	0	0	0	0	0	0	0	0	0	2
Wiqaytna (وقايتنا"Our prevention")	1	0	0	0	1	0	0	0	0	0	0	0	0	0	0	0	0	0	0	0	0	0	0	0	0	0	0	0	0	0	2
NZ COVID Tracer	1	0	0	0	1	0	0	0	0	0	0	0	0	0	0	0	0	0	0	0	0	0	0	0	0	0	0	0	0	0	2
StopKorona!	0	0	0	0	1	1	0	0	0	0	0	0	0	0	0	0	0	0	0	0	0	0	0	0	0	0	0	0	0	0	2
ProteGO Safe	1	1	0	0	1	0	1	0	0	1	0	0	0	0	0	0	0	0	0	0	0	0	0	0	0	1	1	0	0	0	7
TraceTogether	1	1	0	0	1	0	0	0	0	0	0	0	0	0	0	0	0	0	0	0	0	0	0	0	0	0	0	0	0	0	3
Radar COVID	1	0	0	0	1	0	0	0	0	0	0	0	0	0	0	0	0	0	0	0	0	0	0	0	0	0	0	0	0	0	2
Self Shield (Formerly COVID Shield)	0	0	0	0	0	0	1	0	0	0	0	0	0	0	0	0	0	0	0	0	0	0	0	0	0	1	1	0	0	0	3
SwissCovid	1	0	0	0	1	0	0	0	0	0	0	0	0	0	0	0	0	0	0	0	0	0	0	0	0	0	0	0	0	0	2
How We Feel	0	1	0	0	0	0	0	0	0	0	0	0	0	0	0	0	0	0	0	0	0	0	0	0	0	1	1	0	0	0	3
NOVID	1	0	0	0	1	1	0	0	0	0	0	0	0	0	0	0	0	0	0	0	0	0	0	0	0	0	0	0	0	0	3
ProjectCovid	0	1	0	0	0	0	0	0	0	1	1	0	0	1	0	0	0	0	0	0	0	0	0	0	0	0	0	0	0	0	4
Sehet Misr	0	0	0	0	1	0	0	0	0	0	0	0	0	0	0	0	0	0	0	0	0	0	0	0	0	0	0	0	0	0	1
Self-Diagnosis app	0	0	0	0	0	0	0	1	0	0	0	0	0	0	0	0	0	0	0	0	0	0	0	0	0	0	0	0	0	0	1
Self-Quarantine app	0	1	0	0	0	0	0	0	1	0	0	0	0	0	0	0	0	1	0	0	0	0	0	0	0	0	0	0	0	0	3
Private Kit: Safe Paths	0	0	0	0	0	0	0	0	0	0	0	0	0	0	1	0	0	0	0	0	0	0	0	0	0	0	0	0	0	0	1
Coalition App	1	0	0	0	1	0	0	0	0	0	0	0	0	0	0	0	0	0	0	0	0	0	0	0	0	0	0	0	0	0	2
COVID Alert	1	0	0	0	0	0	0	0	0	0	0	0	0	0	0	0	0	0	0	0	0	0	0	0	0	0	0	0	0	0	1
COVIDWISE	1	0	0	0	0	0	0	0	0	0	0	0	0	0	0	0	0	0	0	0	0	0	0	0	0	0	0	0	0	0	1
GuideSafe	1	0	0	0	0	0	0	0	0	0	0	0	0	0	0	0	0	0	0	0	0	0	0	0	0	0	0	0	0	0	1
Covid Watch Arizona	1	0	0	0	0	0	0	0	0	0	0	0	0	0	0	0	0	0	0	0	0	0	0	0	0	0	0	0	0	0	1
BeAware Bahrain	1	0	0	0	1	0	0	0	0	0	0	0	0	0	0	0	0	0	0	0	0	0	0	0	0	0	0	0	0	0	2
CovTracer	0	0	0	0	1	0	0	0	0	0	0	0	0	0	0	0	0	0	0	0	0	0	0	0	0	0	0	0	0	0	1
CareFIJI	0	0	0	0	1	0	0	0	0	0	0	0	0	0	0	0	0	0	0	0	0	0	0	0	0	0	0	0	0	0	1
Shlonik	0	1	1	0	0	0	1	0	1	0	0	0	0	0	0	0	0	0	0	0	0	0	0	0	0	0	1	0	0	0	5
EHTERAZ	0	1	0	0	0	0	0	0	0	0	0	0	0	0	0	0	0	0	0	0	0	0	0	0	0	0	0	0	0	0	1
MorChana	0	0	0	0	1	0	0	0	0	0	0	0	0	0	0	0	0	0	0	0	0	0	0	0	0	0	0	0	0	0	1
E7mi	0	0	0	0	1	0	0	0	0	0	0	0	0	0	0	0	0	0	0	0	0	0	0	0	0	0	0	0	0	0	1
Spectrum	0	1	0	0	0	0	0	0	0	0	0	0	0	0	0	0	0	0	0	0	0	0	0	0	0	0	0	0	0	0	1
Covid AI	0	0	0	0	0	0	0	0	0	0	0	0	0	0	0	0	0	0	0	0	0	0	0	1	0	0	0	0	0	0	1
NIOSH PPE Tracker	0	0	0	0	0	0	0	0	0	0	0	1	0	0	0	0	0	0	0	0	0	0	0	0	0	0	0	0	0	0	1
Pre Work Screen	0	0	0	0	0	0	1	0	0	0	0	0	0	0	0	0	0	0	0	0	0	0	0	0	0	0	0	0	0	0	1
Relief Central Covid-19	0	1	0	0	0	0	0	0	0	0	0	0	0	1	0	0	0	0	0	0	0	0	0	0	0	0	0	0	0	0	2
Osler Covid Learning Centre	0	1	0	0	0	0	0	0	0	0	0	0	0	0	0	0	0	0	0	0	0	0	0	0	0	0	0	0	0	0	1
Hayat Eve Sığar	0	1	0	0	0	0	0	0	0	1	0	0	0	0	0	0	1	0	0	0	0	0	0	0	0	0	0	0	0	0	3

Protect Scotland	1	0	0	0	1	0	0	0	0	0	0	0	0	0	0	0	0	0	0	0	0	0	0	0	0	0	0	0	0	0	2
PathCheck SafePlaces	1	0	0	0	1	0	0	0	0	0	0	0	0	0	0	0	0	0	0	0	0	0	0	0	0	0	0	0	0	0	2
CoronaMelder	1	0	0	0	0	0	0	0	0	0	0	0	0	0	0	0	0	0	0	0	0	0	0	0	0	0	0	0	0	0	1
**Totals**	30	42	5	1	32	3	20	8	6	11	16	2	5	14	7	1	10	1	1	1	1	1	2	6	1	7	16	1	6	1	**258**
